# Epiphytic Bacteria Are Essential for the Production and Transformation of Algae-Derived Carboxyl-Rich Alicyclic Molecule (CRAM)-like DOM

**DOI:** 10.1128/Spectrum.01531-21

**Published:** 2021-10-20

**Authors:** Yang Liu, Jinjun Kan, Chen He, Quan Shi, Yan-Xia Liu, Zhen-Chuan Fan, Jun Sun

**Affiliations:** a Institute of Marine Science and Technology, Shandong University, Qingdao, Shandong, China; b College of Marine Science and Technology, China University of Geosciences (Wuhan), Wuhan, Hubei, China; c Stroud Water Research Center, Avondale, Pennsylvania, USA; d State Key Laboratory of Heavy Oil Processing, China University of Petroleum, Beijing, China; e State Key Laboratory of Food Nutrition and Safety, Institute of Health Biotechnology, Tianjin University of Science and Technology, Tianjin, China; University of Minnesota

**Keywords:** microbial carbon pump (MCP), carboxyl-rich alicyclic molecules (CRAM)-like DOM, *Skeletonema dohrnii*, FT-ICR MS, algae-associated bacteria

## Abstract

The microbial carbon pump (MCP) provides a mechanistic illustration of transformation of recalcitrant dissolved organic matter (DOM) in the ocean. Here, we explored and demonstrated the key roles of algae-associated microorganisms (mainly heterotrophic bacteria) in the production and transformation of carboxyl-rich alicyclic molecule (CRAM)-like DOM through a laboratory experiment involving cultures of Skeletonema dohrnii. Without the participation of the associated bacteria, CRAM-like DOM molecules were not detected via Fourier-transform ion cyclotron resonance mass spectrometry (FT-ICR MS) in algal cultures treated with antibiotics. Similarly, CRAM-like DOM were not detected in cultures of bacteria alone. Our experimental results showed that algae-associated bacteria are important in the process of converting algal-derived organic matter into CRAM-like DOM during S. dohrnii culture. Bacteroidetes (mainly Flavobacteriia) dominated the bacterial community in the stationary and degradation phases, where the predicted metabolic pathways for bacterial assemblages were mainly involved in biosynthesis, metabolism, and degradation. Facilitated by these heterotrophic bacteria, the amount and the chemodiversity of CRAM-like DOM derived from algae varied during the growth and decomposition of algal cells, and CRAM-like DOM were enriched at the later growth stage. The properties and characteristics of these CRAM-like DOM, including molecular weight, double bond equivalent, hydrogen-carbon ratio, carbon-nitrogen ratio, carbon-sulfur ratio, and modified aromaticity index increased with the growth and decay of algal cells, indicating the transformation from active to recalcitrant DOM. In contrast, the organic matter in axenic cultures of *S. dohrnii* mainly existed in the form of particulate organic matters (POM), and small amounts of CRAM-like DOM were detected. This study provides the first laboratory evidence to reveal and confirm the direct involvement of algae-associated microbiomes in the production and transformation of algae-derived refractory DOM, highlighting the significance of these epiphytic bacteria in marine carbon sequestration and global carbon cycling.

**IMPORTANCE** Dissolved organic matter (DOM) serves as a major carbon and nutrient pool in oceans, and recalcitrant DOM are the primary sources for carbon sequestration in depths. Here, we demonstrate the critical roles of algae-associated microorganisms (mainly heterotrophic bacteria) in the transformation of recalcitrant dissolved organic matter through laboratory cultures of a model diatom, Skeletonema dohrnii. Our experimental results showed that in addition to affecting the growth and the physiology of S. dohrnii, algae-associated bacteria are important in processing and converting algal DOM into CRAM-like DOM. Facilitated by the associated bacteria, the amount and the chemodiversity of DOM derived from algae varied during the growth and decomposition of algal cells, and enriched recalcitrant DOM formed in the later growth stage. The properties and diversity of DOM increased with the growth and decay of algal cells, indicating the transformation from active DOM to inert organic matter. Our results confirmed that the direct involvement of algae-associated microbes in the production of CRAM-like DOM. Detailed community structure analysis of the algae-associated bacterial community and its predicted functions confirmed the involvement of certain bacterial groups (e.g., *Flavobacteriia*) in biosynthesis, metabolism, and degradation.

## INTRODUCTION

Oceans are the largest active carbon pool near the Earth’s surface and have significant impacts on global climate change (1, 2). Dissolved organic matter (DOM) is the largest reservoir of organic carbon in the ocean (3), in which more than 90% of total dissolved organic carbon (DOC) can be produced at a rate of 43 Tg per year (4). The majority of DOC is subjected to being modified, forming recalcitrant DOC, and being exported to depth (4). The microbial carbon pump (MCP) provides a mechanistic illustration of the formation of recalcitrant DOM (5, 6), and marine carbon sink processes are dominated by microplankton (7, 8). Phytoplankton or microalgae, including both prokaryotic and eukaryotic cells, are major primary producers in the ocean. Although their biomass accounts for only 1 to 2% of global plants, microalgae contribute 40% of global carbon sequestration each year (9). Microalgae have rich diversity and high biomass in the ocean and can fix carbon dioxide through photosynthesis and convert inorganic carbon into organic carbon. Phytoplankton (especially algae) produce and release DOM into the ambient environment; this is expected to be relatively high-molecular-weight and labile DOM. Labile DOM support high activity of biogeochemical cycling in the surface ocean due to a rapid turnover rate, despite comprising only a small portion of the total DOM pool (10). In addition, microalgae can also consume dissolved nutrients in seawater, regulate the pH of surface seawater, and promote the diffusion of atmospheric carbon dioxide into seawater (11).

Algae and bacteria have coexisted in the ocean for more than 200 million years (12). Most of the DOC and particulate organic carbon (POC) produced by algal metabolism enter the microbial cycle to provide carbon, nutrients, and energy for heterotrophic bacteria (13). In return, these bacteria mineralize and transform 10 to 50% of the photosynthetic products of algae (14) and supply inorganic salts and additional inorganic nutrients for algal growth. This mutually beneficial relationship between algae and bacteria facilitates a continuous DOM regeneration and transformation. The chemical composition and bioreactivity of DOM have changed during/after microbial utilization and processing, resulting in small dissolved molecules (about 10 kDa), which are very resistant to further microbial changes and consumption (15, 16). Throughout the water column in the ocean, the major components of recalcitrant DOM are carboxyl-rich alicyclic molecules (CRAM), which contain a diverse suite of organic compounds with enriched carboxylated and fused alicyclic rings (17). Due to their recalcitrant chemical structure, CRAM compounds can resist rapid microbial degradation, and the decomposition of these compounds depends on distinct bacterial groups and their specific oxidizing capabilities (18). Thus, production of these CRAM-like compounds greatly promotes carbon sequestration in the ocean due to the nature of recalcitrance and chemical complexity. In fact, recalcitrant organic matter can be deposited in sediments or stored in the deep sea for up to thousands of years (19).

Although algae and heterotrophic bacteria have been widely recognized as important drivers for carbon storage in the MCP (5, 20), it is not clear if production of algae-derived CRAM-like DOM relies absolutely on epiphytic bacteria. Previous studies have shown that axenic cultures of multiple species of algae contribute to detectable humic-like DOM in the ocean (21), while others indicated that aggregation of recalcitrant DOM results from microbial processing (mainly bacteria) of planktonic-precursors (22). Incubation experiments have also demonstrated that heterotrophic bacteria in the marine environment can convert glucose, glutamic acid, oligosaccharides, and algae extracts into highly diverse recalcitrant components (16, 23, 24). These exometabolites directly released by bacteria upon utilization of labile substrates ought to have intrinsic properties rendering them resistant to decomposition and therefore allowing them to remain recalcitrant for a long time (16, 21, 25–27). Recently, Fourier-transform ion cyclotron resonance mass spectrometry (FT-ICR MS) has been used in characterizing the composition and structure of DOM molecules in laboratory culture experiments, as well as in environmental samples (28–30). This high-resolution approach provides a great opportunity and makes it possible to monitor the dynamics of DOM production and transformations.

**FIG 1:**
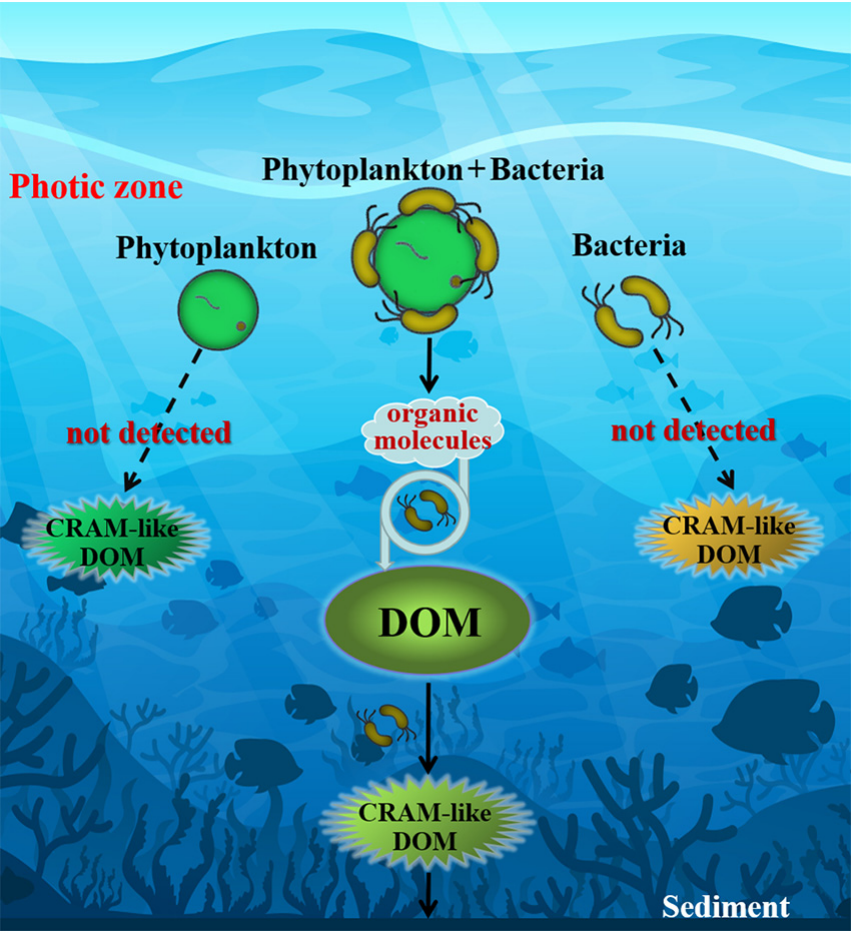
Conceptual model to demonstrate dissolved organic matter (DOM) and CRAM-like DOM production by phytoplankton (*Skeletonema dohrnii*) and bacteria. No CRAM-like DOM was detected via Fourier-transform ion cyclotron resonance mass spectrometry (FT-ICR MS) in treatments with phytoplankton plus antibiotics or with bacteria alone.

In previous work, we have explored the variations of bacterial communities associated with Skeletonema dohrnii cultures via high-throughput sequencing of 16S rRNA genes (31). Our results demonstrate that notable shifts of bacterial compositions occurred during algal growth (31), implying potential roles of these bacteria in algal growth and physiology. For instance, we are not sure if the production of algally produced DOM solely relies on the associated bacteria. Furthermore, we know little about the interactions of the bacterial community with transformation of algal DOM molecules. In this study, we used FT-ICR MS with fluorescence excitation-emission matrix spectroscopy (EEMs) to examine DOM production at different growth stages of *Skeletonema dohrnii* culture. In order to test the roles that associated bacteria play during the process, cultures with and without antibiotics were both examined. DOM productions were monitored at different growth phases, including initial, exponential, stationary, and degradation. Results showed clearly that small amounts of CRAM-like DOM were detected in axenic culture of S. dohrnii, and the amount and the chemodiversity of CRAM-like compounds increased with the growth and degradation of algal cells. The major metabolic pathways of algae-associated bacterial communities were predicted with PICRUSt to demonstrate that microbial communities are closely related to the transformation of DOM and the production of CRAM-like DOM molecules. We conclude that epiphytic bacteria play an indispensable role in algae-derived CRAM-like DOM production (summarized schematically in Fig. 1).

## RESULTS

### Growth of algal and bacterial cultures.

The density of *S. dohrnii* culture (no antibiotics) reached a maximum biomass (9.36 × 10^8^ cells/liter) on day 4 and then, with the senescence of algal cells, the biomass decreased (see Fig. S1 in the supplemental material). The addition of antibiotics led to a longer exponential phase, where the cell density peaked on day 7. Compared to the bacteria-free culture (with antibiotics), algal cells with epiphytic bacteria (no antibiotics) decayed rapidly after the peak, as shown by a rapid drop in cell numbers (Fig. S1). Algae-associated bacteria (alone) grew in artificial sea water (ASW) medium and reached a maximum density at 14.78 × 10^4^ CFU/ml on day 8 (Fig. S1). Fluorescent photographs confirmed the effectiveness of antibiotics (Fig. S2). As expected, no bacterial cells were detected in the algal culture with antibiotics, unlike in the treatment without antibiotics.

### Measurements of DOC and POC.

**FIG 2:**
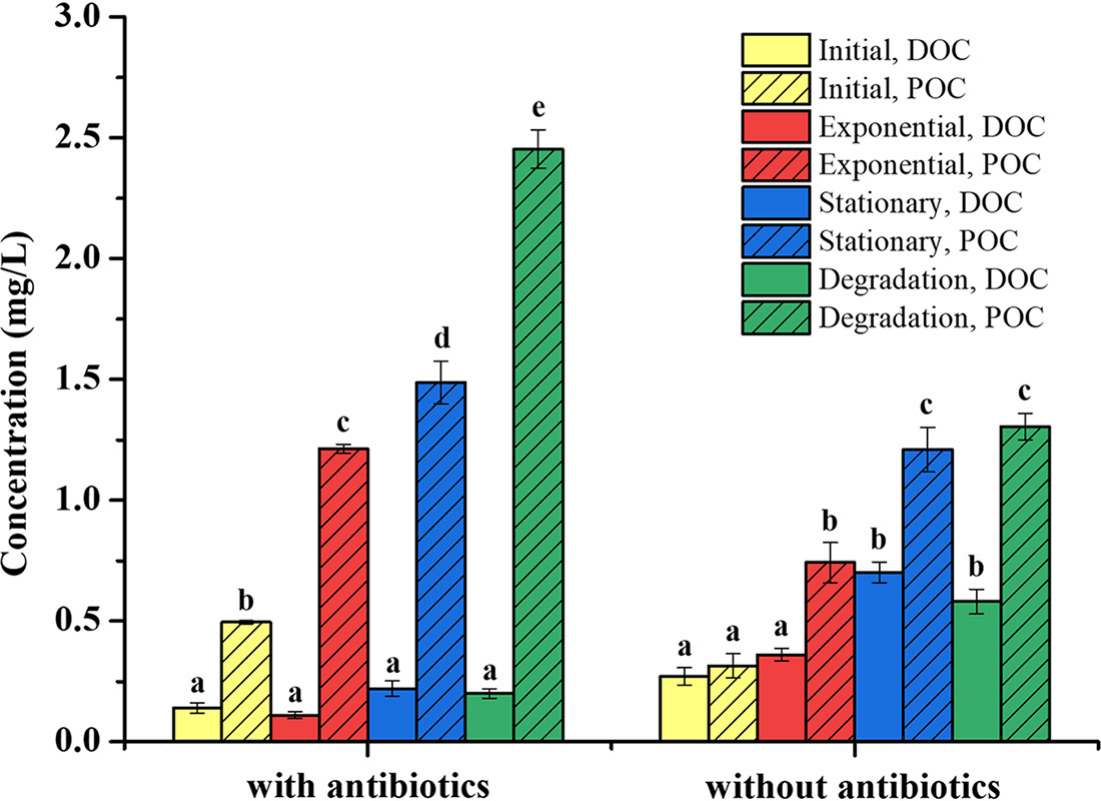
Dissolved organic carbon (DOC) and particulate organic carbon (POC) concentrations for *S. dohrnii* cultures in different growth phases. Samples were collected in initial, exponential, stationary, and degradation phases. All data are presented as mean ± standard error. Values with different letters represent significant difference (*P* < 0.05).

The DOC concentration increased through the stationary phase and decreased in the degradation phase for algal cultures without antibiotics, while it remained consistently low in the treatment with antibiotics, and no significant differences were observed across growth phases (Fig. 2). The DOC concentrations in the stationary and degradation phases for algal cultures without antibiotics were significantly higher than those in the initial and exponential stages. In contrast, the POC concentrations for both cultures (with and without antibiotics) increased during the growth stages, and significantly higher POC concentrations were detected in the treatments with antibiotics than those in the ones without antibiotics (Fig. 2). Algal POC accumulated in the treatment with antibiotics, and the POC concentration reached 2.45 mg/liter in the degradation phase, but the DOC remained low.

### DOM production and characterization with EEMs.

**FIG 3:**
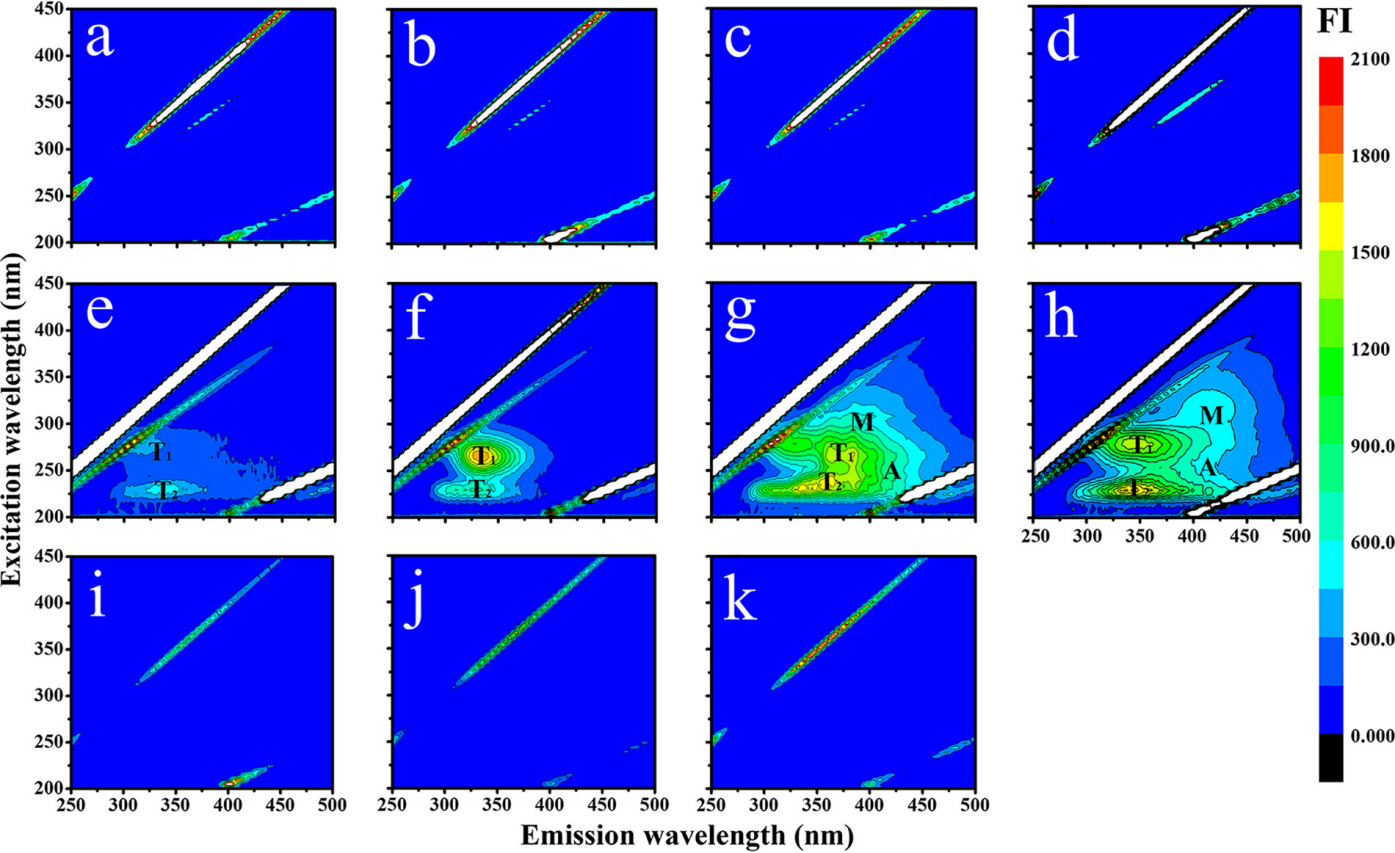
Excitation-emission matrix spectroscopy of DOM fluorescence for *S. dohrnii* cultures in different growth phases (initial, exponential, stationary, and degradation). (a to d) *S. dohrnii* culture with antibiotics; (e to h) *S. dohrnii* without antibiotics; (i to k) bacteria only on day 0, day 6, and day 12, representing the early, exponential, and stationary phases, respectively. Fluorescence intensity (FI) is shown by color gradient. Peaks T_1_ and T_2_ were from tyrosine fluorescence. Peaks M and A represent microbial humic fluorescence and terrigenous humic acid fluorescence, respectively.

No obvious fluorophores were detected in *S. dohrnii* with antibiotics and bacteria alone (Fig. 3a to d and i to k). However, without antibiotics, the fluorescent components from *S. dohrnii* culture gradually increased from the initial to degradation phases (Fig. 3e to h). In the exponential stage, the chromophoric dissolved organic matter (CDOM) was dominated by high-intensity fluorescence of protein-like substances (peaks T_1_ and T_2_) (Fig. 3; excitation/emission at 275/330 to 340 nm and 225/330 to 340 nm). Both peak M (290 to 310/370 to 420 nm) and peak A (230 to 260/380 to 460 nm) started to appear in the stationary phase and remained predominant through cell degradation, while peak M became more distinct and shifted to longer wavelengths (Fig. 3g to h; see also Table S2 in the supplemental material).

### Detailed DOM characterization via FT-ICR MS.

Since DOM in water are polar compounds containing oxygen, they can be effectively ionized and characterized in negative ion mode (32). Similar to EEMs analysis, DOM molecules were only detected in the algal culture without antibiotics. A total of 159 DOM molecules were detected in the initial phase; this number increased to 1,021 in the degradation stage (Table 1). Carbon-hydrogen-oxygen (CHO) compounds were the main DOM molecule detected (56.02% to 91.19%). Relative abundance gradually decreased (from 91.19% to 56.02%) with the growth of *S. dohrnii*, while the number of CHO molecules increased from 145 to 572. In contrast, although less abundant, the relative abundance of carbon-hydrogen-oxygen-nitrogen (CHON) compounds increased with the growth of *S. dohrnii* (from 5.03% to 39.93%). Carbon-hydrogen-oxygen-sulfur (CHOS) compounds were the least abundant, and no significant changes were observed in various growth phases. The average relative average molecular weight (Mass_a_) of DOM molecules were different in different growth phases, namely, 307.45 in the initial phase and increasing to 388.18 in the degradation phase (Table 1). Changes in DOM structures were also distinct from other measurements. For instance, the average oxygen to carbon ratio (O/C_a_) and the average hydrogen to carbon ratio (H/C_a_) were lower in the degradation phase. Meanwhile, C/N_a_ and C/S_a_ increased during the growth and reached their maxima in the degradation phases (13.68 and 16.19, respectively). Similarly, average double bond equivalents (DBE_a_) increased from 5.18 to 7.55 between the initial and degradation phases. Finally, no polycyclic or highly aromatic compounds (modified aromaticity index [AI_mod_] of >0.67) were detected from the initial phase through the stationary phase, but they were present in DOM samples in the degradation phase (Table 1).

**TABLE 1:** Molecular characteristics of DOM for *S. dohrnii* without antibiotics in different growth phases

Growth phase	No. of molecules of:			Total no. of molecules	% of molecules of:			Mass_a_^*a*^	H/C_a_^*b*^	O/C_a_^*c*^	No. of DBE_a_^*d*^	C/N_a_^*e*^	C/S_a_^*f*^	No. (%) of^*g*^:	
	CHO	CHON	CHOS		CHO	CHON	CHOS							0.5 < AI_mod_ < 0.67	AI_mod_ > 0.67
Initial	145	8	6	159	91.19	5.03	3.78	307.45	1.49	0.33	5.18	12.86	14	0 (0)	0 (0)
Exponential	272	127	20	419	64.91	30.31	4.78	352.67	1.43	0.37	6.52	10.69	15.95	3 (0.72)	0 (0)
Stationary	540	311	40	891	60.61	34.90	4.49	367.31	1.41	0.39	6.89	10.23	15.08	13 (1.46)	0 (0)
Degradation	572	408	41	1,021	56.02	39.96	4.02	388.18	1.39	0.36	7.55	13.58	16.19	28 (2.74)	4 (0.39)

a Mass_a_, average molecular weight.

b H/C_a_, average hydrogen to carbon ratio.

c O/C_a_, average oxygen to carbon ratio.

d DBE_a_, average double bond equivalents.

e C/N_a_, average carbon to nitrogen ratio.

f C/S_a_, average carbon to sulfur ratio.

g AI_mod_, modified aromaticity index; 0.5 < AI_mod_ < 0.67, aromatic compounds; AI_mod_ > 0.67, polycyclic aromatic compounds.

**FIG 4:**
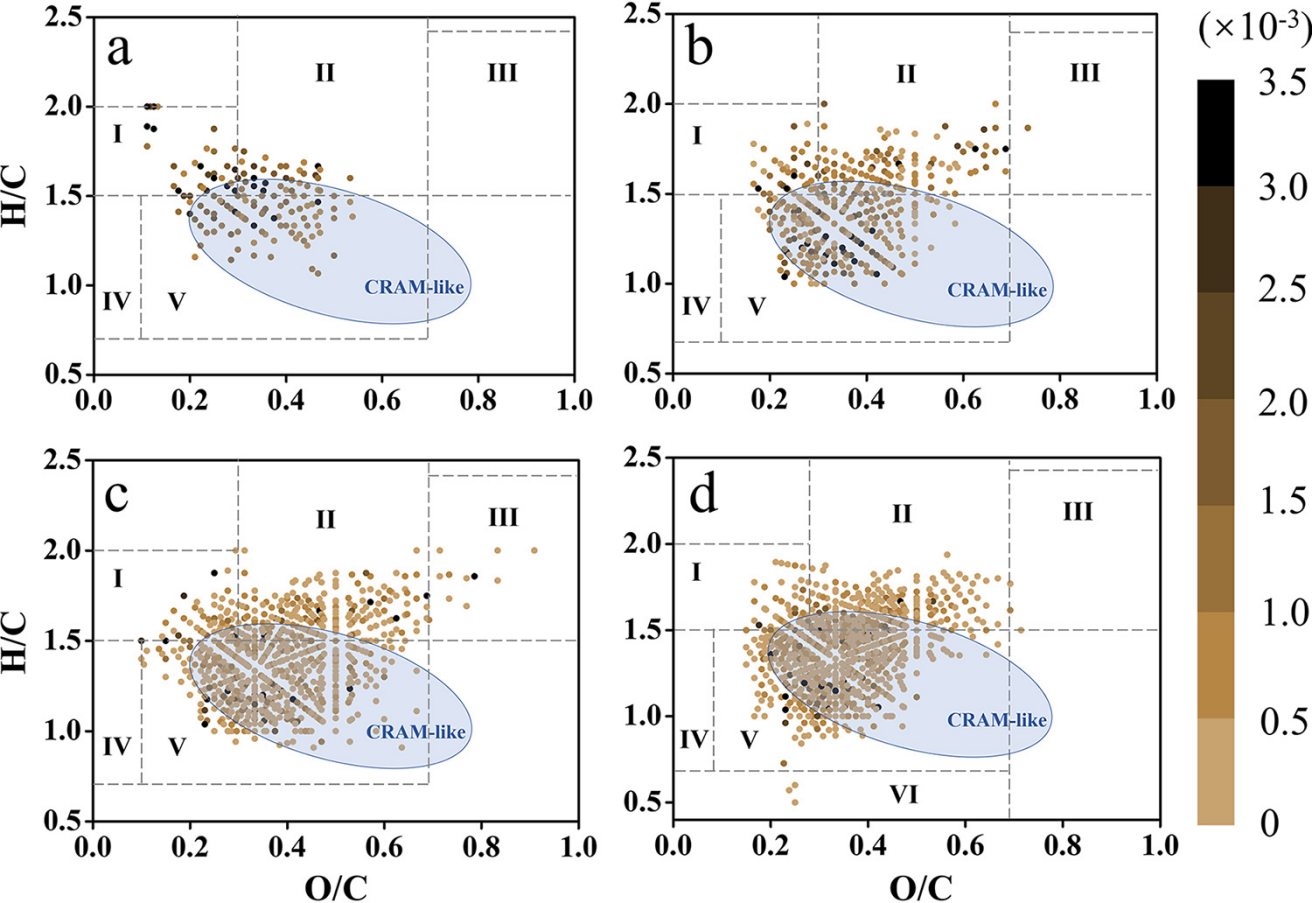
Van Krevelen diagrams of DOM structure and components in different growth phases—initial (a), exponential (b), stationary (c), and degradation (d)—for *S. dohrnii* without antibiotics. DOM molecules were normalized with peak intensity and color coded in conjunction with the Van Krevelen plot. Gradient bar indicates peak intensities. The approximate perimeter of CRAM-like compounds is shown in blue ellipses (DBE/C = 0.30 to 0.68; DBE/H = 0.20 to 0.95; DBE/O = 0.77 to 1.75). DOM compounds were classified based on their H/C and O/C ratios, as follows. Lipid-like region (I), H/C = 1.5 to 2.0, O/C = 0 to 0.3; protein-like region (II), H/C = 1.5 to 2.2, O/C = 0.3 to 0.67; carbohydrate-like region (III), H/C = 1.5 to 2.4, O/C = 0.67 to 1.2; unsaturated hydrocarbon-like region (IV), H/C = 0.7 to 1.5, O/C = 0 to 0.1; lignin-like region (V), H/C = 0.7 to 1.5, O/C = 0.1 to 0.67; condensed aromatic ring structure region (VI), H/C = 0.2 to 0.7, O/C = 0 to 0.67.

Van Krevelen (VK) diagrams further illustrate the dynamic changes of DOM structure and distribution at different growth phases (Fig. 4). Total DOM molecules and CRAM-like compounds increased from initial to degradation phase (Fig. 4a to d). These results confirmed that the numbers and variety of O_x_ compounds (including CHO, CHON, and CHOS) in DOM molecules increased during microalgal growth (Table 1). Based on H/C and O/C ratios, DOM molecules could be classified into different regions (I to VI, listed in Fig. 4). Among them, lignin-like region (V) molecules predominated in DOM materials in all growth phases, and the relative abundance increased from the initial to degradation phases (31.45%, 57.56%, 62.40%, and 64.94% for the respective growth phases). Similarly, DOM molecules belonging to the protein-like region were the second most abundant group, and they increased in the exponential phase and remained abundant until degradation. In addition, molecules with high H/C and low O/C ratios (lipid-like region I) were more abundant in the initial phase, while molecules of the carbohydrate-like region (III) peaked in the stationary phase (Fig. 4).

**FIG 5:**
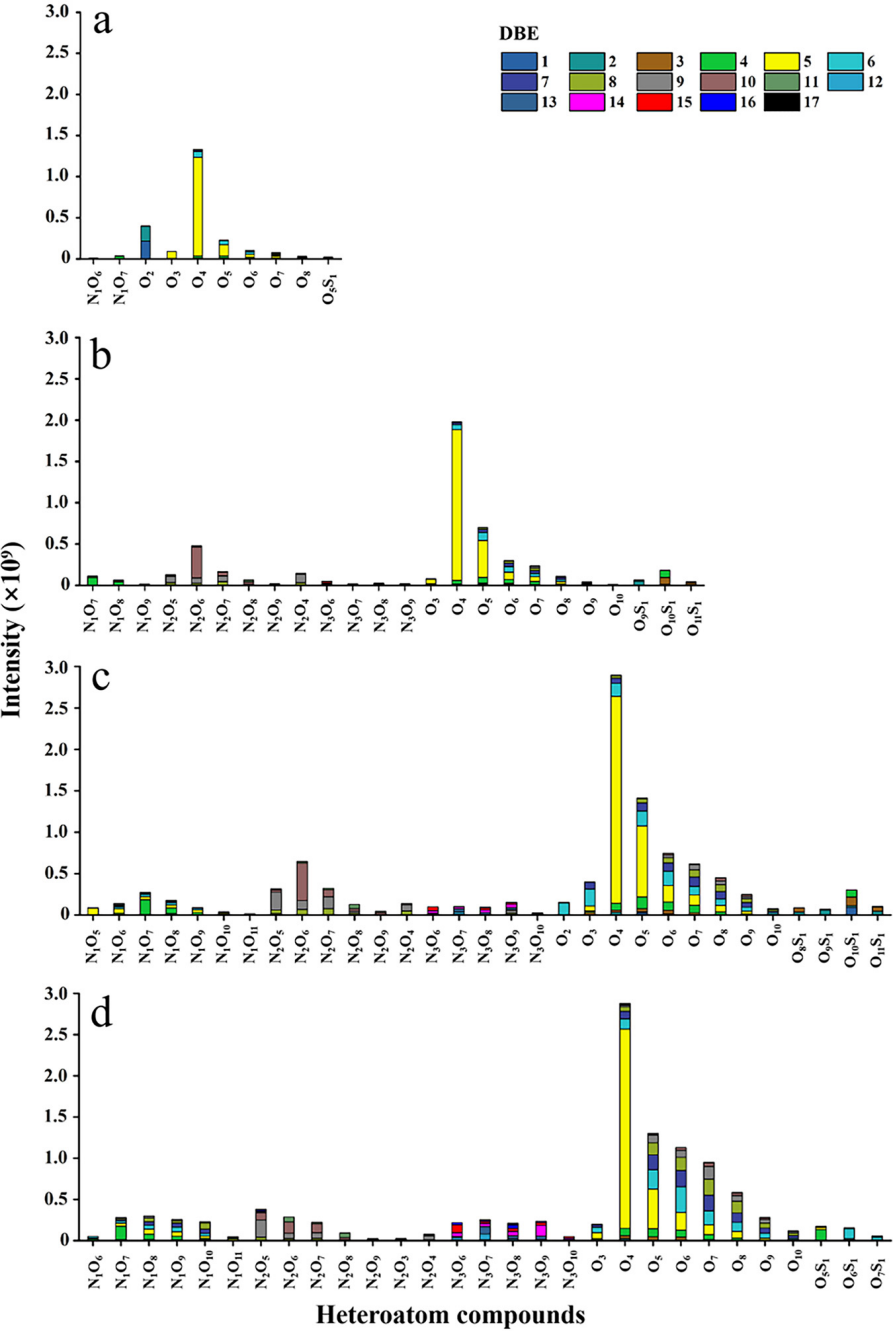
Relative abundance distribution of heteroatom compounds and double bond equivalents (DBE) for solid-phase extraction (SPE) DOM from *S. dohrnii* cultures without antibiotics in different growth phases, as follows: initial (a), exponential (b), stationary (c), and degradation (d).

FT-ICR MS could effectively characterize DOM molecules (C, H, O, N, and S atoms were found in the solid-phase extraction [SPE] DOM samples). Figure 5 shows the relative abundance and distribution of heteroatom compounds, as well as double bond equivalents (DBE) within SPE-DOM. The identifiable peaks in the mass spectrum were all oxygen atom compounds, which were mainly divided into three categories, N_1_O_x_, O_x_, and O_x_S_1_. The O_x_ compounds have obvious advantages regardless of the type of compound or corresponding peak intensity. During the initial phase, 3 major classes (N_1_O_x_, O_x_, and O_x_S_1_) and 10 minor classes of compounds were found (Fig. 5a). Furthermore, 5 major classes (N_1_O_x_, N_2_O_x_, N_3_O_x_, O_x_, and O_x_S_1_) and 24, 31, and 29 minor classes of compounds were found in the exponential, stationary, and degradation phases, respectively. In brief, DBE distribution of heteroatom compounds increased from 9 to 17 with algal growth. These results indicated that compounds were mainly low in condensation (Fig. 5). The O_x_ compound species were the most abundant throughout growth, and peaks corresponding to the 4-oxygen (O_4_) compounds were the highest, respectively accounting for 56.97%, 39.14%, 27.61%, and 25.88% of total peak intensities (Fig. 5). In contrast, the relative intensities of nitrogen and sulfur-containing compounds were low, and most of them comprised less than 1% of total intensity (Fig. S4a to d). The N-1 and N-3 compounds did not show any distinguishable patterns between growth phases (Fig. S4a and c), while the H/C ratios of N-2 and S-1 formulas were significantly different between the exponential/stationary and degradation phases (*P* < 0.05; Fig. S4b and d), indicating DOM structural changes upon cell death and degradation.

### Associated microbial community structure, indicator ASVs, and predicted functions at different growth stages.

**FIG 6:**
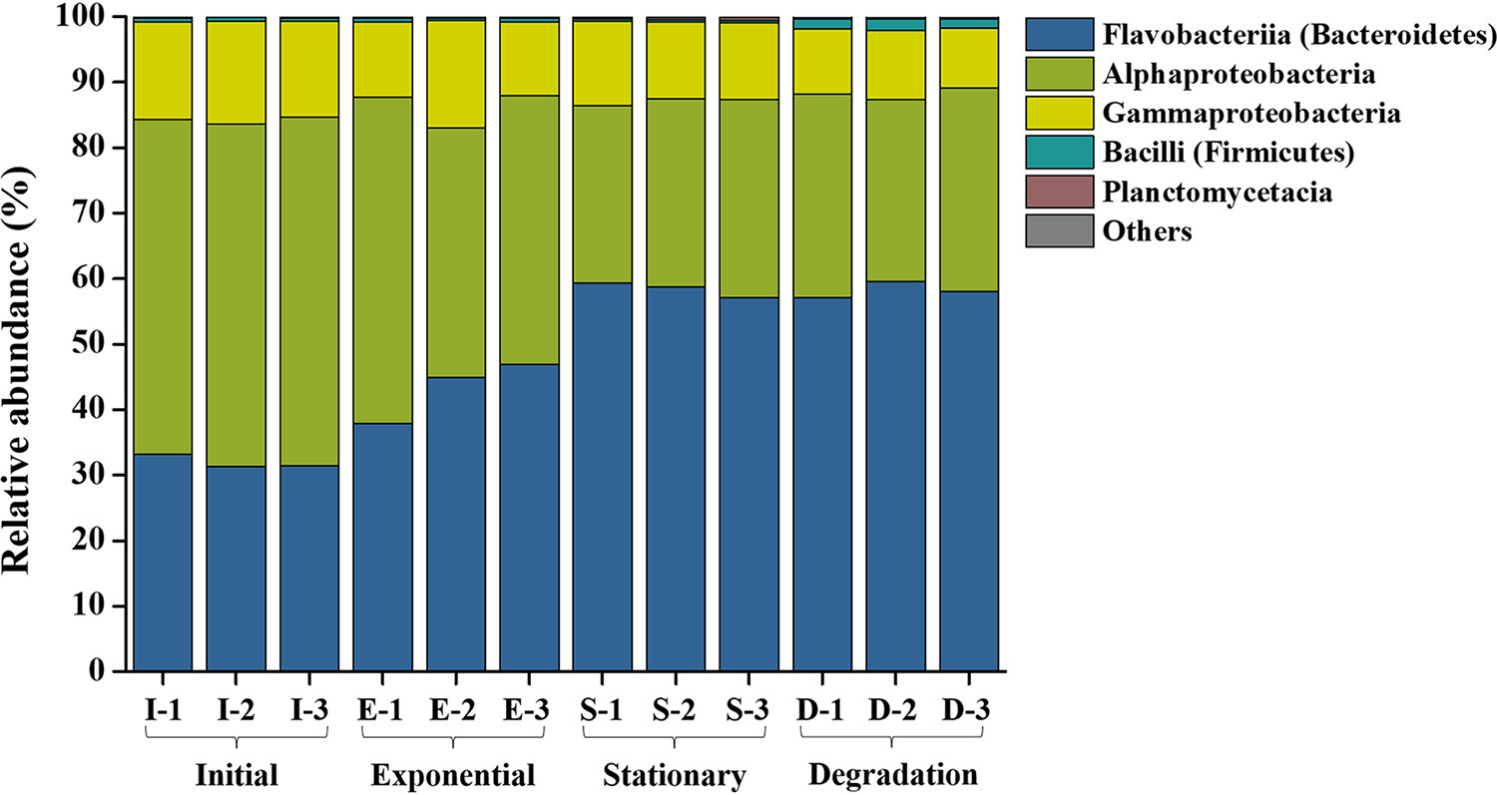
Epiphytic bacterial community structures of the *S. dohrnii* cultures in different growth phases. Three biological replicates were used for each growth phase. The corresponding SRA accession numbers for initial (I-1, I-2, and I-3), exponential (E-1, E-2, and E-3), stationary (S-1, S-2, and S-3), and degradation (D-1, D-2, and D-3) phases are SRX7222072 (I-1), SRX7222073 (I-2), SRX7222110 (I-3), SRX7222074 (E-1), SRX7222075 (E-2), SRX7222076 (E-3), SRX7222084 (S-1), SRX7222085 (S-2), SRX7222086 (S-3), SRX7222097 (D-1), SRX7222098 (D-2), and SRX7222099 (D-3).

Detailed microbial community structures indicated that a clear composition shift occurred during the algal growth. Proteobacteria (Alphaprotebacteria and Gammaproteobacteria) decreased, while Bacteroidetes (Flavobacteriia) and Firmicutes (Bacilli) increased from the initial to the later growth stages (Fig. 6). The indicator amplicon sequence variants (ASVs) also suggested community change, and the top 15 indicator ASVs in four different growth phases are listed in Table S3. In early stages (initial and exponential), *Alphaproteobacteria* (mainly Rhodobacteraceae) and *Gammaproteobacteria* (Alteromonadaceae) dominated the indicator ASVs, but more indictor ASVs from *Flavobacteriia* (e.g., Flavobacteriaceae, Cryomorphaceae), and different *Gammaproteobacteria* (e.g., Solimonadaceae and Alcanivoracaceae, Piscirickettsiaceae, and Oceanospirillaceae) were identified in later growth phases (stationary and degradation) (Table S3).

**FIG 7:**
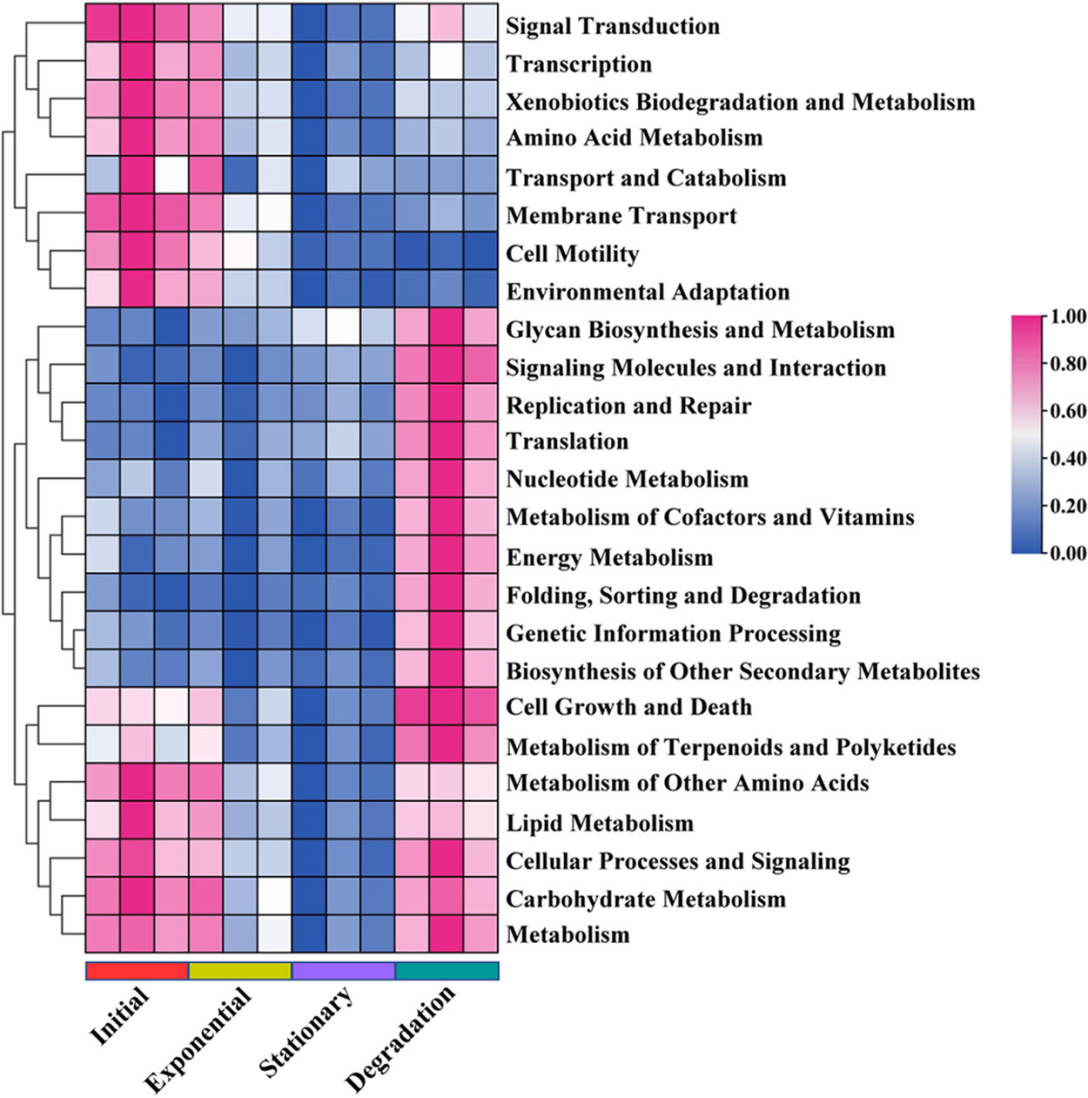
Relative abundance of predicted Kyoto Encyclopedia of Genes and Genomes (KEGG) metabolic pathways during the four growth phases. Scale bar represents row Z-scores of normalized expression values that increase from blue to pink (range, 0 to 1).

The metabolic functions (top 25 pathways) of epiphytic bacterial communities and their relative abundances were predicted by PICRUSt and are shown in Fig. 7. The predicted pathways in early stages are primarily involved in “signal transduction,” “transcription,” “biodegradation and metabolism,” “transport and catabolism,” “cell motility,” and “environmental adaptation” (Fig. S3a and b). In contrast, “glycan biosynthesis and metabolism,” “signaling molecules and interaction,” “replication and repair,” “nucleotide metabolism,” “energy metabolism,” and “folding, sorting and degradation” are enriched in the later stages of growth (Fig. S3c and d). Interestingly, “cell growth and death,” lipid metabolism,” metabolism pathways for “carbohydrate,” “terpenoids and polyketides,” and “lipid” were predicted to be abundant in both early and late growth phases (Fig. 7; see also Fig. S3).

## DISCUSSION

Our results confirmed that algae-associated bacteria are important in CRAM production, whereas only small amounts of CRAM-like compounds were detected in cultures of either bacteria alone or *S. dohrnii* alone (with antibiotics) (Fig. 1). As the algae grew, unstable compounds/metabolites from *S. dohrnii* were likely utilized and processed by heterotrophic microorganisms, so the molecular structure and diversity of DOM changed and became more resistant to further degradations (16). As a major contributor to marine primary productivity in oceans, microalgae (e.g., *S. dohrnii*) secrete various metabolites (such as carbohydrates, amino acids, enzymes, organic acids, vitamins, promoting factors, and inhibiting factors) to surrounding environments (33–35). These metabolites are precursors of CRAM-like compounds and are further processed by heterotrophic microorganisms while promoting the accumulation of CRAM-like DOM (3, 36). Heterotrophic microbes are both decomposers and producers of organic carbon (16, 37). It has been experimentally demonstrated that heterotrophic bacteria can consume DOM even at very low concentrations (nanomolar) and generate recalcitrant compounds (16, 38, 39). For instance, heterotrophic bacteria in the ocean can use labile DOC produced by phytoplankton and convert them to recalcitrant DOC (40). The high-resolution FI-ICR MS data in this study clearly demonstrated the DOM transformation and structural changes during algae growth and the role of epiphytic microorganisms in the DOM generation process.

Detailed characterization of the epiphytic bacterial communities associated with *S. dohrnii* indicates that heterotrophic bacterial groups such as Bacteroidetes (e.g., Aquibacter spp. and Fluviicola spp. from *Flavobacteriia*) are likely involved in the consumption and transformation of algae-derived DOM (reference 31 and this study). *Bacteroidetes*, which include *Flavobacteriia*, comprise a well-known bacterial group specialized in degrading high-molecular-weight organic compounds (41). In marine environments, *Bacteroidetes* are enriched on organic matter particles (42) and are capable of consuming polymers rather than monomers (43). They are also commonly found to be abundant during and following algal blooms (44), suggesting their close association with algal cell growth and degradation. It has been reported that *Flavobacteriia* are involved in degrading the organic matter that is produced by algae (41), as evidenced by the increase in the relative abundance of *Flavobacteriia* in this study (Fig. 6). In fact, a previous study demonstrated that a single bacterial species cannot degrade high-molecular-weight DOM compounds completely, implying that collaborative efforts of multiple species are required (45). On the other hand, the relative abundances of predicted functional pathways such as the main metabolic process (carbohydrate, lipid, and energy metabolism) are greatly enriched at later stages of algal growth (Fig. 7), suggesting that the associated bacterial consortia can utilize a broad spectrum of DOM compounds (46) and transform the DOM compounds to recalcitrant forms (e.g., CRAM-like molecules).

In order to eliminate or limit bacterial contamination, a mixture of antibiotics is normally used to treat the algal cultures (47–49). These antibiotics are broad-spectrum antibiotics that interfere with cell wall synthesis (e.g., penicillin) or protein processes (e.g., chloramphenicol and kanamycin) for prokaryotic cells only (50–52). In general, the broad-spectrum antibiotics are effective against several Gram-positive and most Gram-negative bacteria and might have minimal impact on algal growth, depending on the individual species (48). In our study, higher cell density occurred in bacteria-free cultures of *S. dohrnii* than in nonaxenic cultures (treated with antibiotics), and this effect was likely attributed to reduced light availability and/or nutrient competition (53, 54). However, no impact on algal metabolisms is expected (55, 56). For instance, physiological tests on multiple species of algal cultures showed that mixed antibiotics have little or no effect on oxygen evolution, chlorophyll fluorescence kinetics, or pigment content under different light conditions (57). Similarly, Jones et al. (58) demonstrated no changes in cytology, pigmentation, or any other growth characteristics for two diatoms under different combinations of antibiotics. Therefore, we believe that the antibiotics applied in this study do not have a significant impact on algal metabolism, especially in terms of organic matter production.

Our results indicated that the DOM produced by *S. dohrnii* (with antibiotics) remained constantly low and was not detected by FT-ICR-MS, which is in contrast to a previous study in which production of fluorescent dissolved organic matter was detected by EEMs in axenic cultures of Skeletonema (18). Another study also claimed that autochthonous processes contributed to at least a portion of chromophoric DOM (CDOM) in the ocean, but that the majority of recalcitrant DOC accumulation was facilitated by bacterial processing (16, 19, 59–61). It is likely that the majority of the organic matter produced by algae is present primarily as particulate organic matter (Fig. 2, POC) (62, 63). Under the action of bacteria, POM is continuously transformed from coarse POM to fine POM to high-molecular-weight DOM and finally to recalcitrant high-molecular-weight DOM (64). However, without the participation of bacteria, the majority of POM is not able to be further converted to recalcitrant DOM but is maintained as particulate forms (65). In addition, low abundances of microbes and no DOM were detected in the bacteria-only treatment, which is likely due to the limitation of substrates in artificial seawater (21, 22, 66). In brief, this observation might be related to the low concentration of microbially derived DOM compared to that of background DOM.

In our study, EEMs patterns of *S. dohrnii*-derived DOM were similar to those from three other diatoms (Skeletonema grethae, Leptocylindrus hargravesii, and Coscinodiscus sp.) and one haptophyte (Phaeocystis globosa), with peak T (T_1_ and T_2_) being the most prominent (19). However, the DOM composition data from FT-ICR MS were distinct from that detected in natural seawater. In general, thousands of DOM molecules can be detected in natural seawater and exhibit a Gaussian distribution (normal distribution) (67–69), but the DOM molecules from this study demonstrated no obvious regularity. Due to the single species of microphytoplankton (*S. dohrnii*), the numbers and types of DOM molecules detected are limited and simple compared to those in natural environments. However, the major compounds detected in this study are consistent with previous observations in rivers and lakes (67–69), in which O_x_ compounds, mainly carbon, hydrogen, and oxygen (CHO) molecules were the major components (Table 1; 56.02% to 91.19%). These CHO molecules likely result from algal materials processed by heterotrophic microorganisms and contribute to carbon sequestration (21). The majority of CHO molecules retrieved in this study were lignin-like substances (Fig. 4) (H/C,0.7 to 1.5; O/C, 0.1 to 0.67), suggesting their algae and cell wall origins (70–72). Lignins, most of which belong to the CRAM-like category, have higher chemical stability and resistance to microbial degradation (Fig. 4). Last, we noticed that among O_x_ compounds, low-oxygen compounds were predominant in DOM molecules (73), but high-oxygen compounds were not detected, which was likely due to the inhibition of effective ionization by the high content of low-oxygen compounds (74).

The molecular composition and chemodiversity changes in DOM during different growth phases of *S. dohrnii* suggest that DOM molecules were progressively transformed from protein-like substances (initial and exponential phases) to humic-like substances (stationary and degradation phases) (Fig. 3e to h). In addition, FT-ICR MS data showed that the average molecular weight gradually increased from 307.45 in the initial phase to 388.18 in the degradation phase (Table 1), indicating the aging of DOM from initial to the degradation phase. However, the average molecular weight range in this study was lower than those reported by other studies in natural ocean environments (75, 76), and the discrepancy may be attributed to the difference between the sample and the substrate and to instrument variations (77). The DBE_a_ also increased from the initial to degradation phases (Table 1), indicating that the degree of unsaturation increased in SPE-DOM samples associated with *S. dohrnii* culture (78, 79). It is likely that the microorganisms transform the DOM to molecules containing more oxygen atoms or heteroatoms during the repeated use of organic carbon and form condensed aromatics with higher-energy bonds (16, 80). Furthermore, the shifts of heteroatom compounds and nitrogen/sulfur-containing compounds clearly demonstrated the DOM structure changes during algal growth (Table 1; see also Fig. S4 in the supplemental material). For instance, significantly lower H/C ratios of S-1 compounds were observed in the degradation phase than in the exponential/stationary phases (Fig. S4d and h). Considering that the molecular formulas of heteroatoms (e.g., H/C, C/N_a_, and C/S_a_) have been widely accepted and serve as important indicators for DOM characteristics and lability (81), DOM stability was demonstrated by DOM structure with less hydrogen in later growth phases, which was likely due to processes such as “hydrogen-dissipating” oxidation (65). Interestingly, N-2 molecules exhibited an opposite trend from that of S-1 compounds, where higher H/C ratios occurred in the degradation phase (Fig. S4b and f). We speculate that these compounds were processed and reused by microorganisms, while DOM lost carbon (therefore higher H/C) and was converted into compounds containing more oxygen or other heteroatoms. These compounds were highly cyclized DOM with high molecular binding energy (i.e., DBE), indicating a conversion of active DOM to inert organic matter (16, 78). This is consistent with the finding that DOM in the degradation phase was more recalcitrant than DOM in the initial phase (82).

AI_mod_ is another indicator for DOM characteristics, although the AI_mod_ measurements are a relatively conservative way to identify aromatic materials (83). In this study, polycyclic aromatic compounds (AI_mod_ > 0.67) were detected during the degradation phase, and a relatively large number of aromatic compounds (0.5 < AI_mod_ < 0.67) were also produced in the degradation phase (Table 1). During algal growth, both aromatic compounds are potentially degraded by associated microbial communities (84). Furthermore, heterotrophic bacteria can also secrete alginolytic compounds to lyse and disrupt microalgae cell and their metabolites, including carbohydrates, proteins, lipids, polysaccharides, and other macromolecular compounds (85). The allelopathic activity of secreted compounds, mode of action, and disruption efficiency depend on the nature of the compound released and differ with the targeted microalga, cell wall rigidity, and composition (85). In return, algae-derived DOM molecules play a role in shaping the community structure and the diversity of related heterotrophic bacteria (86, 87). Obviously, these processes and interactions are also influenced by physicochemical associations of organic molecules, metabolic energy yields, and environmental conditions, which deserve future investigation.

### Conclusions.

Overall, our study demonstrates that algae-associated bacteria play an important role in the production of algae-associated DOM and its transformation. CRAM-like DOM were not detected in bacteria-free algal cultures, suggesting these bacteria, mainly *Bacteroidetes* and *Proteobacteria*, are needed in DOM transformation. Different amounts and chemodiversity of CRAM-like compounds were derived in different growth phases of *Skeletonema dohrnii*, and these compounds were more enriched in the later growth phase. These results further corroborate the significance of heterotrophic bacteria and their potential impacts on marine carbon sequestration via the MCP. In the future, we will continue exploring how algae-associated microorganisms manipulate global carbon cycling and impact climate change.

## MATERIALS AND METHODS

### *Skeletonema* culture conditions and growth.

Microalgal *Skeletonema* spp. are red tide algal species that are widely distributed in offshore China. *Skeletonema dohrnii* was originally isolated from the central Yellow Sea, China (88). *S. dohrnii* was cultivated and transferred with artificial seawater (ASW) medium in transparent conical flasks at 25°C. The light intensity was maintained at 100 μmol photons · m^−2^ ·s^−1^ and the photoperiod consisted of a 14 h:10 h light-dark cycle. In order to regularly monitor the algal growth, 100 μl of culture solution was mounted onto a blood cell counting slide, and algae cells were numerated with a microscope (BX51; Olympus, Japan).

The following three treatments were set up in this study: (i) *S. dohrnii* cultured in ASW with antibiotics, (ii) *Skeletonema dohrnii* cultured in ASW without antibiotics, and (iii) algae-associated bacteria alone grown in ASW (bacteria only). For treatments i and ii, *S. dohrnii* cells were inoculated into 2,000- ml transparent conical flasks containing 1,800 ml of ASW medium, and the initial algal concentration was around 4.25 × 10^8^ cells/liter. Algal growth curves were monitored by microscopic counts (89). For treatment iii, *S. dohrnii* cells were cultivated in ASW medium as in treatment ii, and after reaching the exponential growth phase, algal cells were removed with centrifugation. At this point, the algae-associated bacteria were retained in the medium, meaning that the leftover medium containing bacteria continued until the end of the experiment. The light intensity and photoperiod were the same as those described above. All experiments were carried out in triplicate.

### Antibiotic selection and testing.

The antibiotics selected for this experiment were penicillin, chloramphenicol, and kanamycin sulfate. An orthogonal experiment was conducted on dosages for antibiotics at 0, 50, 100, 200, and 400 mg/liter. The effectiveness of antibiotics and the sterility were tested with a plating method (90) and acridine orange fluorescence staining (91). Based on testing results, the final concentration and combination were 200 mg/liter each for penicillin, chloramphenicol, and kanamycin sulfate (see Table S1 in the supplemental material; no bacteria were detected with penicillin, chloramphenicol, and kanamycin sulfate at 200 mg/liter, and therefore this combination was used in the experiment). In addition, axenic diatoms obtained from the antibiotic treatment were subcultured in order to eliminate the effects of antibiotics.

### Measurements of DOC and POC.

To prevent carbon contamination, all glass materials used for sample collection and storage were acid washed, rinsed with Milli-Q water, and precombusted (450°C for 6 h). The samples (20 ml) were directly collected into 40-ml glass vials and immediately stored at −20°C for subsequent dissolved organic carbon (DOC) analysis. DOC was measured using a total organic carbon analyzer (TOC-3100; Germany). To determine the particulate organic carbon (POC) for *S. dohrnii* culture, cells were harvested using precombusted (450°C for 6 h) GF/F filters. The samples were acidified with 200 μl of 0.2 N HCl and dried overnight in an oven at 60°C before analysis with an elemental analyzer (ECS4010; Costech, Italy).

### Excitation-emission matrix spectroscopy.

DOM samples (10 ml) were filtered through a 0.7-μm combusted GF/F filter into precombusted (450°C for 6 h) glass vials. Excitation-emission matrix spectra were measured using a F-7100 fluorescence spectrophotometer (Hitachi, Tokyo, Japan) with excitation (Ex) from 250 to 450 nm, and emission (Em) from 250 to 500 nm at 5-nm and 2-nm intervals, respectively. The slit widths were 5 nm for both Ex and Em, using a scanning speed of 12,000 nm/min at 700 V. Fluorescence results were concatenated into EEM spectra for visualization as contour plots. The software Origin 8.5 was employed for handling EEMs information.

### Sample preparation and solid-phase extraction.

In brief, the experimental process is summarized as follows: living and nonliving particulate materials were removed by filtration through a 0.2-μm polycarbonate membrane (Millipore, USA). Filtrate was stored in 250-ml borosilicate bottles (precombusted at 450°C for 6 h). The solid-phase extraction (SPE) PPL cartridges (Bond Elut PPL, 200 mg; Agilent, America) were rinsed with three volumes (3 ml) of pure methanol (liquid chromatography-mass spectrometry [LC-MS] grade) and with acidified (pH = 2, HCl) ultrapure water (Milli-Q water) immediately before use. The filtrate went through Teflon tubes to the extraction column and PPL cartridges by natural gravity.

Due to the potential interference with DOM ionization, inorganic salts were removed by a previously described method (92). After the sample completely passed through the PPL cartridge, inorganic salt in the PPL cartridge was washed with 9 ml acidified (pH = 2, HCl) and ultrapure water (Milli-Q water) followed by nitrogen blowing to remove water. DOM from the PPL cartridges were eluted with 3 ml LC-MS grade methanol and stored at −18°C in the dark for later analysis.

### ESI FT-ICR MS analysis.

Electrospray ionization (ESI), invented by Fenn et al. (93), is currently the most suitable ionization method for analyzing the molecular composition of DOM. The solubility of DOM represents strong molecular polarity, which can be easily ionized in an ESI source. A small amount of heteroatom polar compounds can be selectively ionized from a high-concentration complex hydrocarbon matrix. Analysis of DOM sample was performed using an Apex-Ultra FT-ICR mass spectrometer (Bruker, America) equipped with 9.4 T magnetic fields. Every DOM sample was dissolved in a methanol solution and, at the same time, a syringe pump was used to inject an electrospray source at 180 μl/h. The instrument detection parameter settings were emission voltage at 3 kV, capillary column voltage at 4 kV, and capillary column end voltage at −320 kV. The delay time and data size were set to 1.0 ms and 2 million words, respectively. After internal calibration, the mass accuracy changed to ≤0.2 ppm for singly charged ions throughout an extensive *m/z* range (i.e., *m/z* 200 to 800) to ensure an excessive accuracy in elemental formula assignments in this mechanistic observation. Methanol was used as a blank control prior to analysis of the samples. Data processing was conducted using Compass Data Analysis version 4.1 (Bruker, Bremen, Germany). Chemical formulas were batch calculated from their exact masses using a software tool developed in house (mass error window ≤ 500 ppb). When multiple assignments were possible for a single *m/z* value, the formula was assigned based on homologous series (94). The generated formulas were validated by setting reasonable chemical constraints, namely signal-to-noise ratio (S/N) ≥ 6, O/C ratio ≥ 1, H/C ratio ≥ 0.3, and the following element counts: ^12^C ≤ 100, ^13^C ≤ 2, ^1^H ≤ 100, ^14^N ≤ 5, ^16^O ≤ 20, ^32^S2, and ^34^S ≤ 1. The final chemical formula assignments were assigned to groups containing CHO, CHNO, or CHOS chemical compositions, which were used to reconstruct the group-selective mass spectra. The double bond equivalents (DBE) relevant to the execution of elemental formula assignment were calculated for each component (95, 96).

Molecular compositions of the DOM were visualized in Van Krevelen (VK) diagrams. Various molecular formulas were plotted based on their H/C and O/C atomic ratios so that the unique H/C and O/C values for each DOM formula can be shown as previously described (91, 92). Moreover, the DOM molecular function groups can be classified by modified aromaticity index (AI_mod_) (83),

$$AI_{\mathrm{mod}}=\frac{1+C-0.5O-S-0.5(N+P+H)}{C-0.5O-N-S-P}$$

where C, H, N, S, and P represent the numbers of carbon (C), hydrogen (H), nitrogen (N), sulfur (S), and phosphorus (P) atoms in each DOM formula, respectively. Based on AI_mod_, DOM can be roughly divided into polycyclic aromatic compounds (AI_mod_ > 0.67) and aromatic compounds (0.5 < AI_mod_ < 0.67) (83). It should be noted that this categorization of compound groups from FT-ICR MS is only based on the elemental ratios and AI_mod_ and thus bears the limitation that categorization is not absolute since FT-ICR MS does not provide structural information for DOM.

### Statistical analysis and microbial data.

In this study, a paired *t* test was implemented via SPSS software (version 26.0) wherever applicable. Statistically significant differences were defined at a confidence level of 95% with a *P* value of <0.05. Detailed analysis of bacterial compositions based on high-throughput sequencing data was reported elsewhere (31), and the 16S rRNA gene sequences were deposited in the GenBank database under accession numbers SRX7222072 to SRX7222110. Details for DNA extraction, high-throughput sequencing, ASV generation, and sequence analysis can be found in a previous publication (31). Based on the detailed DOM characterization in this study, 12 samples (NCBI SRA accession numbers SRX7222072 to SRX7222076, SRX7222084 to SRX7222086, SRX7222097 to SRX7222099, and SRX7222110) were selected and used for further analyses, as follows. (i) Indicator amplicon sequence variants (ASVs) associated with *S. dohrnii* in different growth phases were determined by Dufrene-Legendre indicator species analysis using the “multipatt” function (97), and (ii) predictive functional profiling and metabolic pathways were conducted using PICRUSt analysis (98).

### Data availability.

This study addresses a unique and important research question regarding the significance roles of algae-associated bacteria in production and transformation of DOM molecules. A subset of high-throughput sequences from a previous publication (31) was used in this study. The data set and primary studies are acknowledged and cited in this article. These sequence data have been deposited in GenBank with public accession numbers, so the data are findable, accessible, interoperable, and reusable.
