# Supplementary material for: Epiphytic Bacteria Are Essential for the Production and Transformation of Algae-Derived Carboxyl-Rich Alicyclic Molecule (CRAM)-like DOM
Source: Microbiol Spectr. 2021 Oct 20;9(2):e01531-21. doi: 10.1128/Spectrum.01531-21 (PMC8528127; doi:10.1128/Spectrum.01531-21)
Supplement: SUPPLEMENTAL FILE 1 — Supplemental material. Download SPECTRUM01531-21_Supp_1_seq4.pdf, PDF file, 2.2 MB [file spectrum01531-21_supp_1_seq4.pdf]

Journal: *Microbiology Spectrum*

Supplementary information for

Epiphytic bacteria are essential for the production and transformation of algae-derived carboxyl-rich alicyclic molecules (CRAM)-like DOM

Yang Liu[1,2], Jinjun Kan[3]*, Chen He[4], Quan Shi[4], Yan-Xia Liu[5], Zhen-Chuan Fan[5], Jun Sun[2]*

[1] Institute of Marine Science and Technology, Shandong University, Qingdao, Shandong 266237, China.

[2] College of Marine Science and Technology, China University of Geosciences (Wuhan), Wuhan, Hubei 430074, China.

[3] Stroud Water Research Center, 970 Spencer Road, Avondale, PA 19311, USA.

[4] State Key Laboratory of Heavy Oil Processing, China University of Petroleum, Beijing 102249, China.

[5] State Key Laboratory of Food Nutrition and Safety, Institute of Health Biotechnology, Tianjin University of Science and Technology, Tianjin 300457, China.

Corresponding e-mail: jkan@stroudcenter.org and phytoplankton@163.com

Contents of this file:

- **Figures: 4**
- **Tables: 3**
- **References: 4**

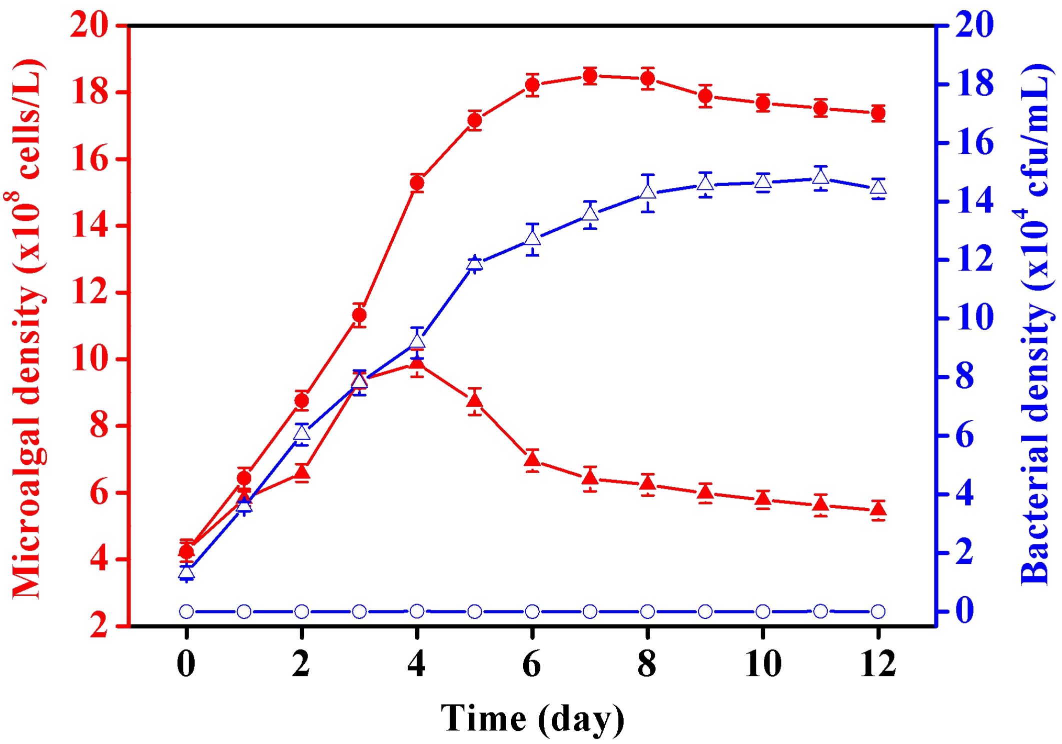

Fig. S1 Growth curves for *S. dohrnii* cultures (red) and bacteria (blue). ○, with antibiotics; △, without antibiotics.

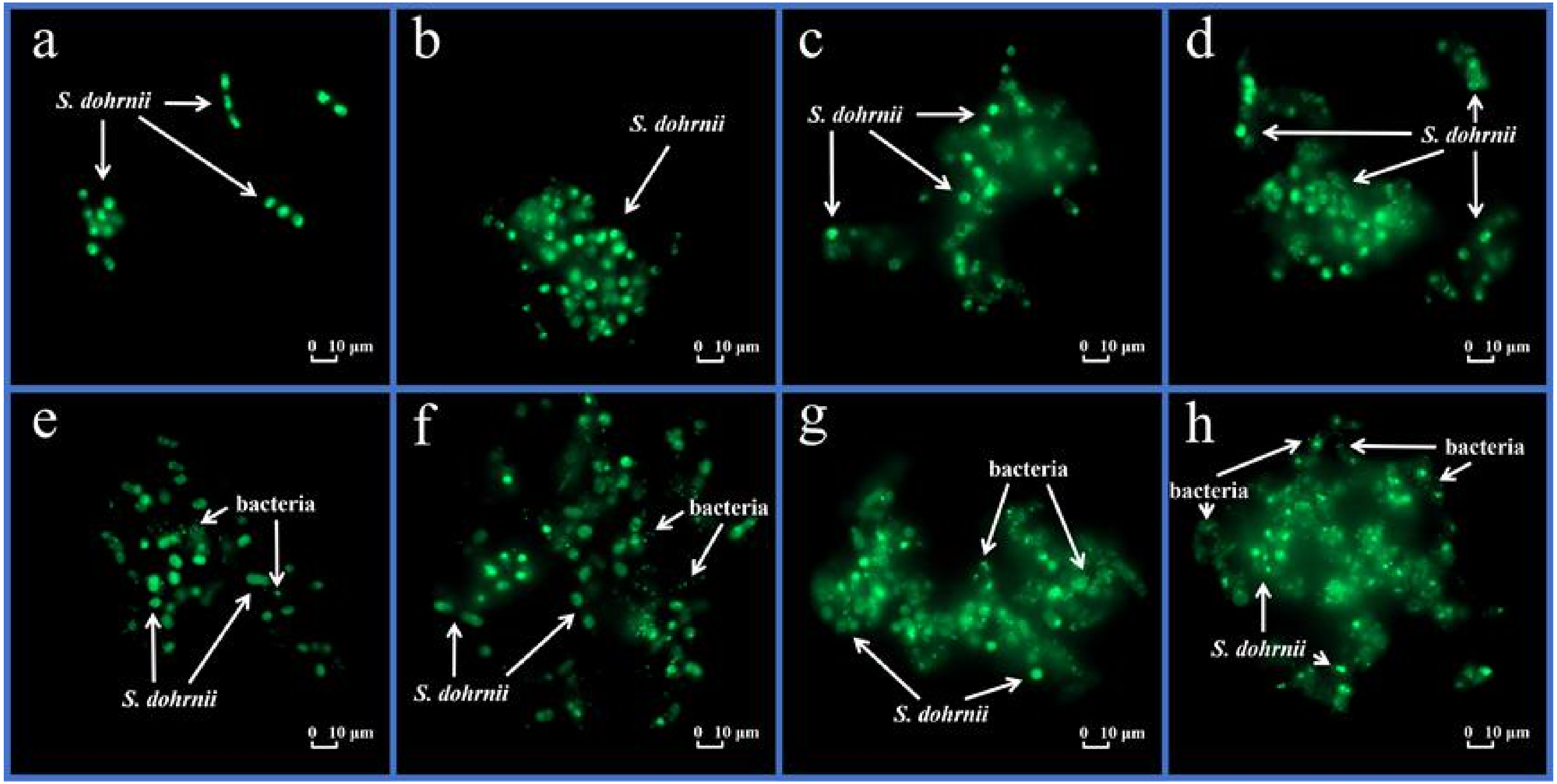

Fig. S2 Fluorescence photographs of *S. dohrnii* and bacteria at different growth phases with (a-d) and without antibiotics (e-h). a-d and e-h represented initial, exponential, stationary and degradation respectively.

a

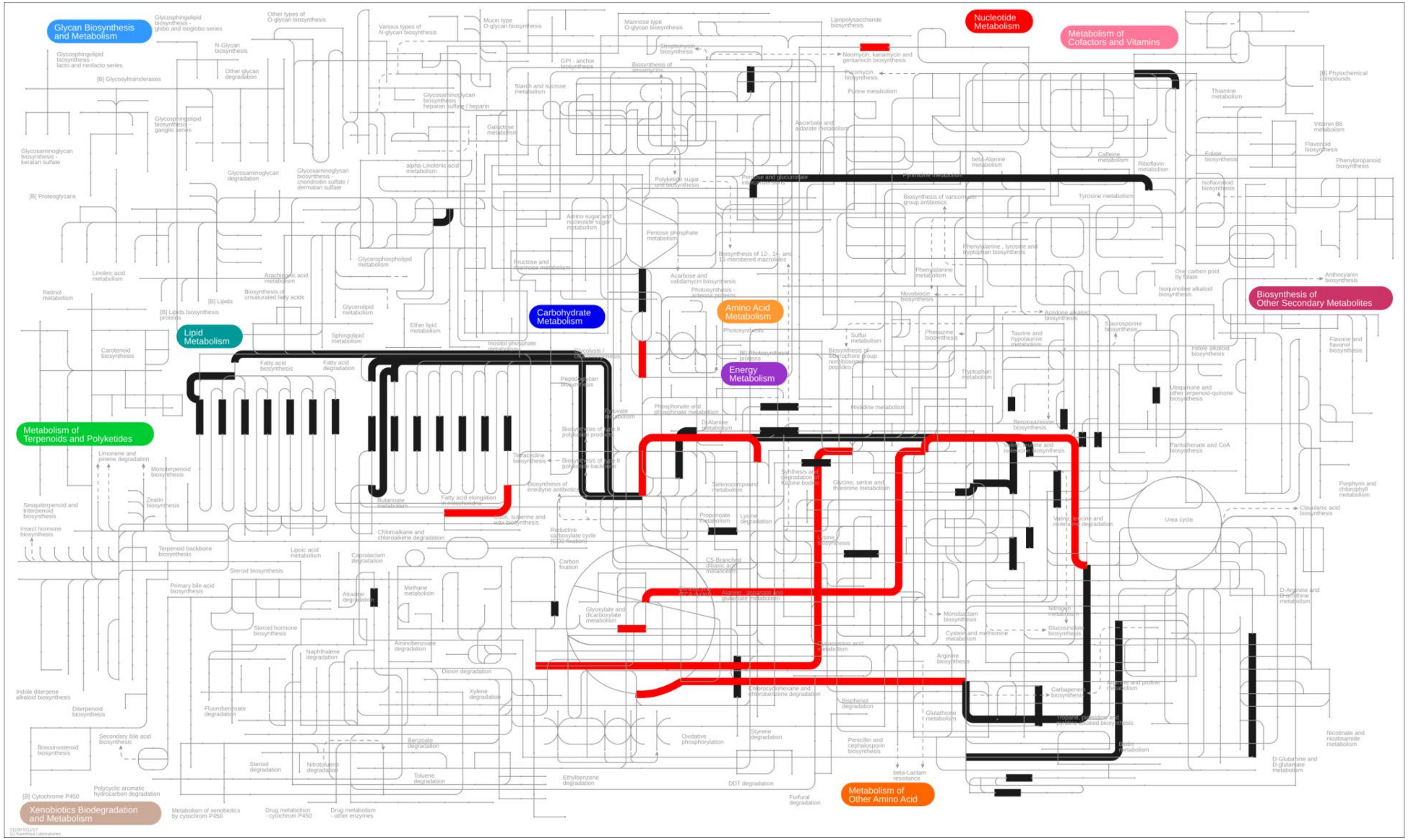
Glycan Biosynthesis and Metabolism
Nucleotide Metabolism
Metabolism of Cofactors and Vitamins
Biosynthesis of Other Secondary Metabolites
Carbohydrate Metabolism
Amino Acid Metabolism
Lipid Metabolism
Energy Metabolism
Metabolism of Terpenoids and Polyketides
Metabolism of Other Amino Acid
Xenobiotics Biodegradation and Metabolism

b

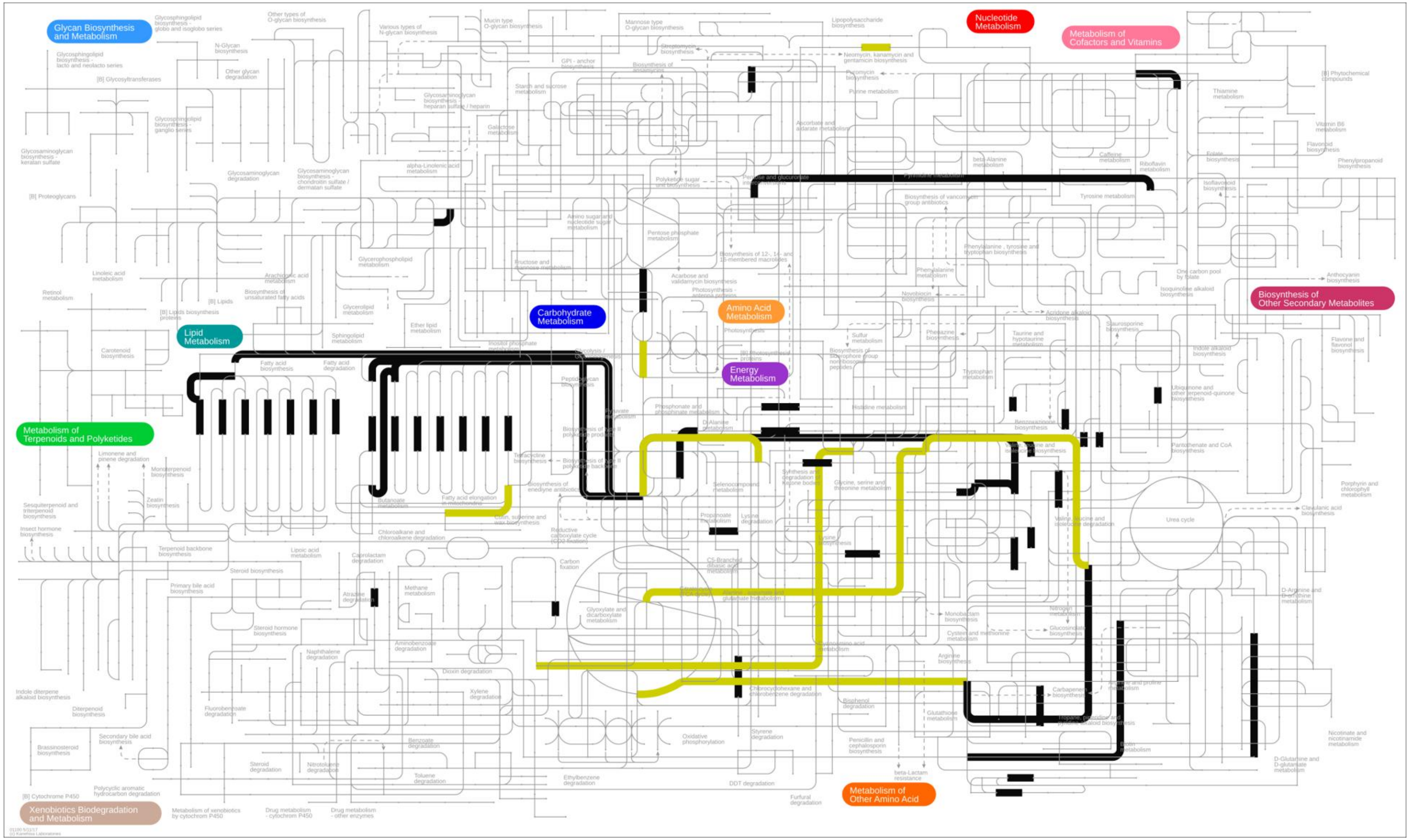
Glycan Biosynthesis and Metabolism
Nucleotide Metabolism
Metabolism of Cofactors and Vitamins
Biosynthesis of Other Secondary Metabolites
Carbohydrate Metabolism
Amino Acid Metabolism
Lipid Metabolism
Energy Metabolism
Metabolism of Terpenoids and Polyketides
Metabolism of Other Amino Acid
Xenobiotics Biodegradation and Metabolism
Urea cycle

c

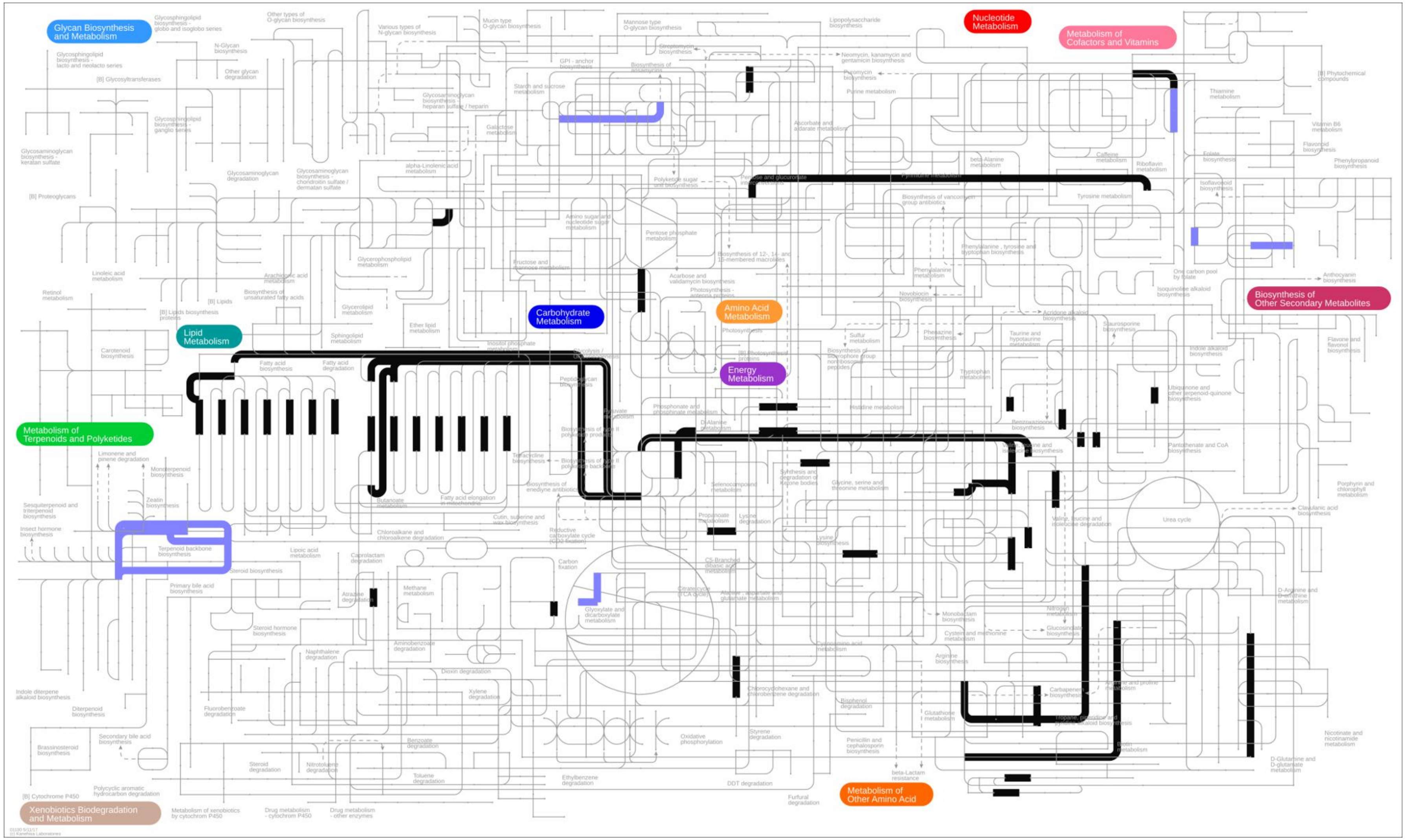
Glycan Biosynthesis and Metabolism
Nucleotide Metabolism
Metabolism of Cofactors and Vitamins
Biosynthesis of Other Secondary Metabolites
Amino Acid Metabolism
Carbohydrate Metabolism
Lipid Metabolism
Energy Metabolism
Metabolism of Terpenoids and Polyketides
Xenobiotics Biodegradation and Metabolism
Metabolism of Other Amino Acid

d

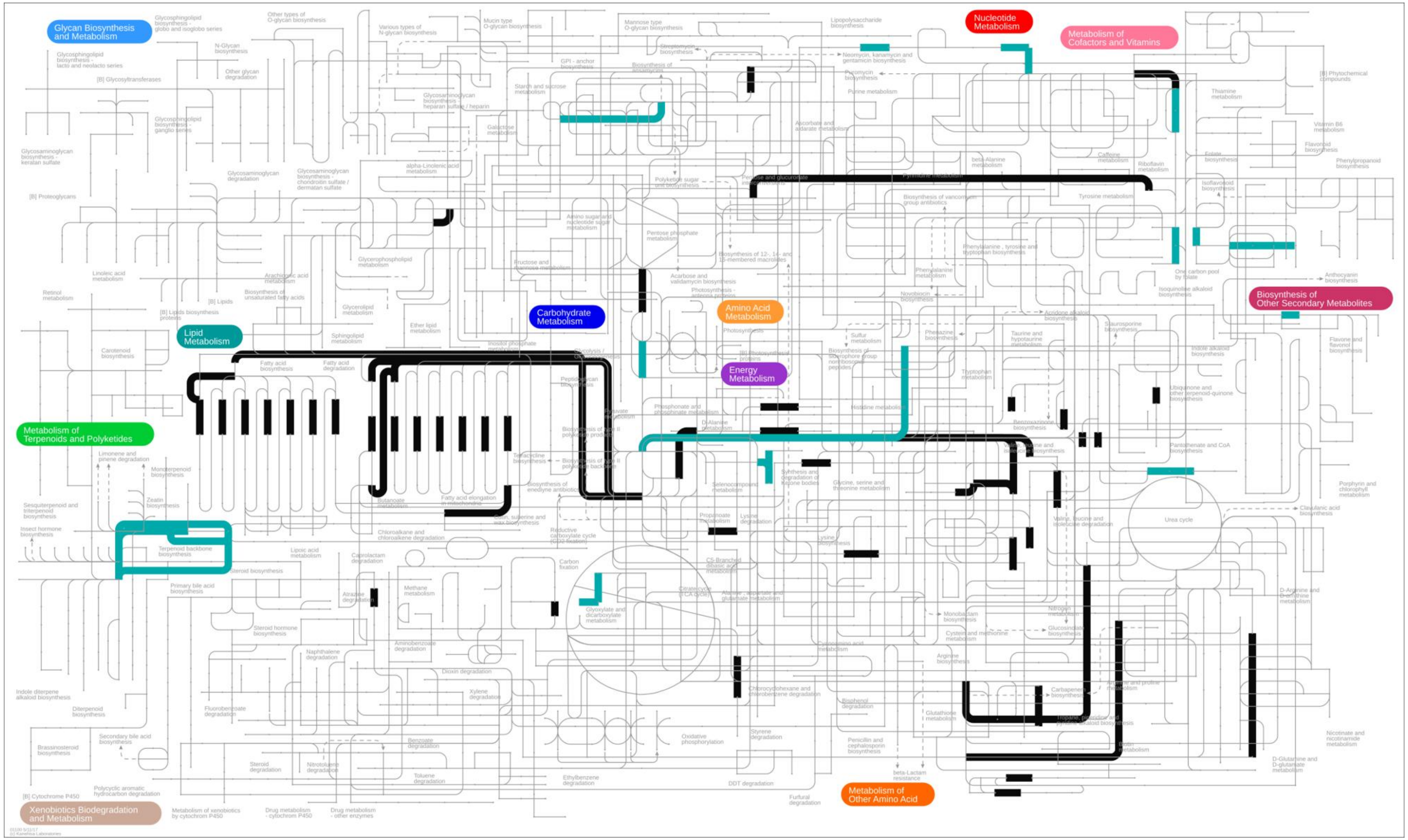

Glycan Biosynthesis and Metabolism
Nucleotide Metabolism
Metabolism of Cofactors and Vitamins
Biosynthesis of Other Secondary Metabolites
Carbohydrate Metabolism
Amino Acid Metabolism
Lipid Metabolism
Energy Metabolism
Metabolism of Terpenoids and Polyketides
Metabolism of Other Amino Acid
Xenobiotics Biodegradation and Metabolism

Fig. S3 Predicted metabolic pathway of associated bacterial communities (log_2 RPKM values greater than 15) at different algal growth phases (a-d represented initial, exponential, stationary and degradation, respectively). Pathways were generated using the iPath 3.0 (1) based on KEGG annotation of genes detected from sequencing. RPKM, read number per kb of each coding sequence, per million mapped sequences. The colors black represents the common metabolic pathway, while red, yellow, purple and green represent the unique metabolic pathways at different growth phases.

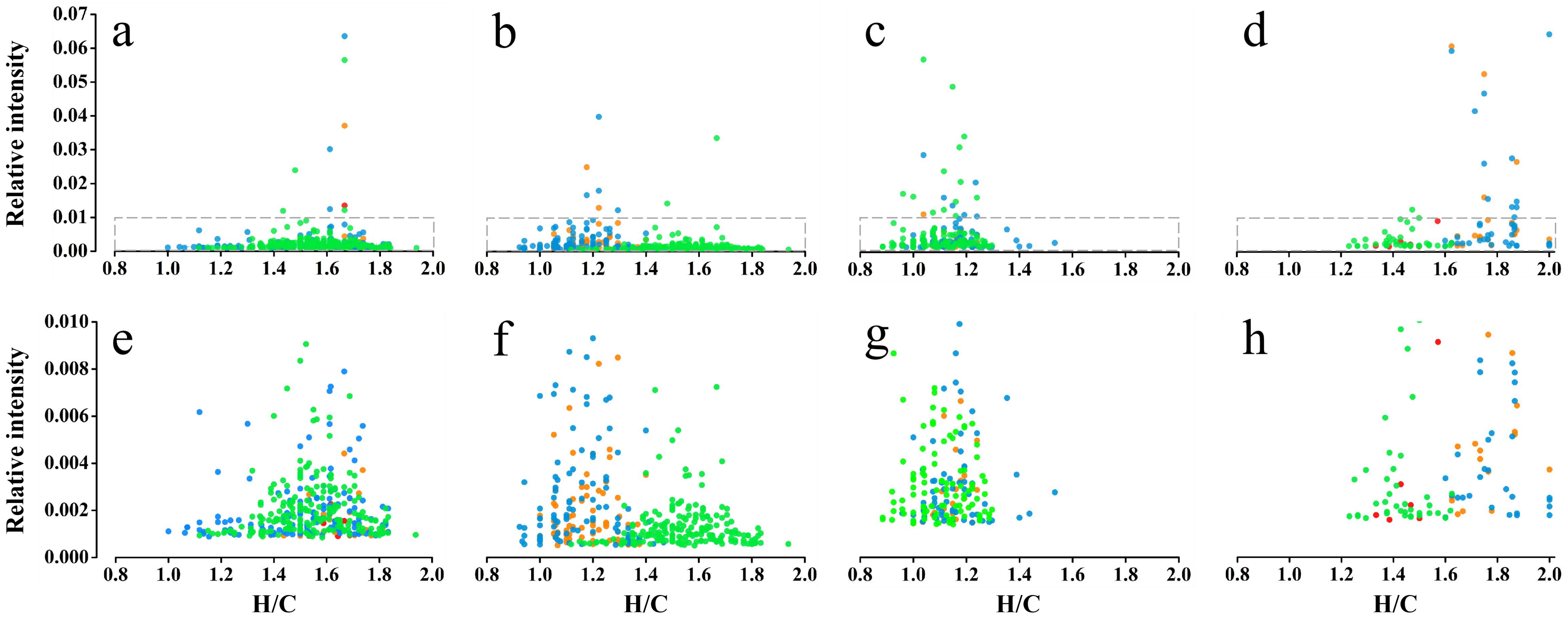

Fig. S4 Relative intensity and distribution of nitrogen and sulfer-containing DOM compounds at different growth phases (●, initial; ●, exponential; ●, stationary; ●, degradation). **a**, formulas with one nitrogen atom (N=1); **b**, formulas with two nitrogen atoms (N=2); **c**, formulas with three nitrogen atom (N=3); **d**, formulas with one sulfur atom (S=1). Enlarged images of **a**-**d** (gray dotted box) were shown in **e**-**h**, respectively.

Table S1. Orthogonal testing for combinations of antibiotics used in the experiment

Antibiotics concentration (mg/L)	0	50	100	200	400
Antibiotics selection	(×)	a, (×)<br>b, (×)<br>c, (×)<br>a+b, (×)<br>b+c, (×)<br>a+c, (×)<br>a+b+c, (×)	a, (×)<br>b, (×)<br>c, (×)<br>a+b, (×)<br>b+c, (×)<br>a+c, (×)<br>a+b+c, (×)	a, (×)<br>b, (×)<br>c, (×)<br>a+b, (×)<br>b+c, (×)<br>a+c, (×)<br>**a+b+c, (√)**	a, (×)<br>b, (×)<br>c, (×)<br>a+b, (×)<br>b+c, (×)<br>a+c, (×)<br>a+b+c, (√)

a, penicillin; **b**, chloramphenicol; **c**, kanamycin sulfate; ×, bacteria detected; and √, no bacteria.

Table S2. Peaks of EEMs fluorescence attributed to different sources of organic matter

Peak	Excitation wavelength (Ex, nm)	Emission wavelength (Em, nm)	Description	Comparison with previous studies
A	230-260	380-460	Humic-like	Terrestrial and marine humic-like, Peak A, Ex/Em: 235/404, (2)
M	290-310	370-420	Humic-like	Microbial processing of organic matter, Peak M, Ex/Em: 310/414, (3)
T_1, T_2	225, 275	330-340	Protein-like	Tryptophan-like, Peak T, Ex/Em:: 225(275)/340, (4)

Table S3. Top 15 bacterial indicator ASVs at different growth phases in the *S. dohrnii* cultures

Growth phases	ASV_ID	Phylum	Class	order	family	genus	species	Indicator value index	P value
Initial	ASV_2	Proteobacteria	Gammaproteobacteria	Alteromonadales	Alteromonadaceae	/	/	0.171	0.003
Initial	ASV_6	Proteobacteria	Alphaproteobacteria	Rhodobacterales	Rhodobacteraceae	/	/	0.223	0.007
Initial	ASV_34	Proteobacteria	Alphaproteobacteria	Rhodobacterales	Rhodobacteraceae	/	/	0.165	0.018
Initial	ASV_42	Firmicutes	Bacilli	Bacillales	Bacillaceae	Bacillus	Bacillus_sp._M71_S33	0.144	0.004
Initial	ASV_100	Proteobacteria	Alphaproteobacteria	Rhodobacterales	Rhodobacteraceae	/	/	0.162	0.017
Initial	ASV_244	Proteobacteria	Alphaproteobacteria	Rhodobacterales	Rhodobacteraceae	Sulfitobacter	/	0.165	0.004
Initial	ASV_311	Proteobacteria	Alphaproteobacteria	Rhodobacterales	Rhodobacteraceae	/	/	0.185	0.029
Initial	ASV_315	Proteobacteria	Alphaproteobacteria	Rhodobacterales	Rhodobacteraceae	/	/	0.166	0.023
Initial	ASV_327	Proteobacteria	Alphaproteobacteria	Rhodobacterales	Rhodobacteraceae	/	/	0.181	0.027
Initial	ASV_340	Proteobacteria	Alphaproteobacteria	Rhodobacterales	Rhodobacteraceae	/	/	0.135	0.004
Initial	ASV_368	Proteobacteria	Gammaproteobacteria	Alteromonadales	Alteromonadaceae	Marinobacter	/	0.345	0.002
Initial	ASV_375	Proteobacteria	Alphaproteobacteria	Rhodobacterales	Rhodobacteraceae	Marivita	/	0.217	0.039
Initial	ASV_393	Proteobacteria	Alphaproteobacteria	Rhodobacterales	Rhodobacteraceae	/	/	0.215	0.035
Initial	ASV_398	Proteobacteria	Alphaproteobacteria	Rhodobacterales	Rhodobacteraceae	/	/	0.294	0.007
Initial	ASV_427	Proteobacteria	Alphaproteobacteria	Rhodobacterales	Rhodobacteraceae	/	/	0.343	0.007
Exponential	ASV_6	Proteobacteria	Alphaproteobacteria	Rhodobacterales	Rhodobacteraceae	/	/	0.163	0.007
Exponential	ASV_34	Proteobacteria	Alphaproteobacteria	Rhodobacterales	Rhodobacteraceae	/	/	0.164	0.018
Exponential	ASV_100	Proteobacteria	Alphaproteobacteria	Rhodobacterales	Rhodobacteraceae	/	/	0.191	0.017
Exponential	ASV_311	Proteobacteria	Alphaproteobacteria	Rhodobacterales	Rhodobacteraceae	/	/	0.177	0.029
Exponential	ASV_315	Proteobacteria	Alphaproteobacteria	Rhodobacterales	Rhodobacteraceae	/	/	0.175	0.023
Exponential	ASV_327	Proteobacteria	Alphaproteobacteria	Rhodobacterales	Rhodobacteraceae	/	/	0.165	0.027

Exponential	OUT_337	Proteobacteria	Gammaproteobacteria	Alteromonadales	Alteromonadaceae	Marinobacter	/	0.109	0.007
Exponential	ASV_340	Proteobacteria	Alphaproteobacteria	Rhodobacterales	Rhodobacteraceae	/	/	0.135	0.004
Exponential	ASV_356	Proteobacteria	Alphaproteobacteria	/	/	/	/	0.105	0.003
Exponential	ASV_366	Bacteroidetes	Flavobacteriia	Flavobacteriales	Flavobacteriaceae	Aquibacter	/	0.155	0.044
Exponential	ASV_380	Proteobacteria	Alphaproteobacteria	Rhodobacterales	Rhodobacteraceae	/	/	0.234	0.045
Exponential	OUT_389	Proteobacteria	Alphaproteobacteria	Rhodobacterales	Rhodobacteraceae	/	/	0.126	0.009
Exponential	ASV_393	Proteobacteria	Alphaproteobacteria	Rhodobacterales	Rhodobacteraceae	/	/	0.169	0.035
Exponential	ASV_398	Proteobacteria	Alphaproteobacteria	Rhodobacterales	Rhodobacteraceae	/	/	0.171	0.007
Exponential	ASV_427	Proteobacteria	Alphaproteobacteria	Rhodobacterales	Rhodobacteraceae	/	/	0.231	0.007
Stationary	OUT_3	Bacteroidetes	Flavobacteriia	Flavobacteriales	Flavobacteriaceae	Aquibacter	/	0.096	0.015
Stationary	ASV_4	Bacteroidetes	Flavobacteriia	Flavobacteriales	Cryomorphaceae	Fluviicola	/	0.114	0.018
Stationary	ASV_21	Proteobacteria	Alphaproteobacteria	Caulobacterales	Hyphomonadaceae	Maricaulis	/	0.107	0.003
Stationary	ASV_72	Proteobacteria	Gammaproteobacteria	Cellvibrionales	Spongiibacteraceae	Spongiibacter	Ambiguous_taxa	0.151	0.001
Stationary	ASV_100	Proteobacteria	Alphaproteobacteria	Rhodobacterales	Rhodobacteraceae	/	/	0.126	0.017
Stationary	ASV_308	Proteobacteria	Alphaproteobacteria	/	/	/	/	0.121	0.012
Stationary	ASV_316	Bacteroidetes	Flavobacteriia	Flavobacteriales	Cryomorphaceae	Fluviicola	/	0.121	0.020
Stationary	ASV_327	Proteobacteria	Alphaproteobacteria	Rhodobacterales	Rhodobacteraceae	/	/	0.102	0.027
Stationary	ASV_330	Bacteroidetes	Flavobacteriia	Flavobacteriales	Flavobacteriaceae	Aquibacter	/	0.113	0.014
Stationary	ASV_337	Proteobacteria	Gammaproteobacteria	Alteromonadales	Alteromonadaceae	Marinobacter	/	0.120	0.007
Stationary	ASV_340	Proteobacteria	Alphaproteobacteria	Rhodobacterales	Rhodobacteraceae	/	/	0.117	0.004
Stationary	ASV_344	Proteobacteria	Alphaproteobacteria	Rhodobacterales	Rhodobacteraceae	Marinovum	alpha_proteobacterium_GMD21D01	0.121	0.041
Stationary	ASV_360	Proteobacteria	Alphaproteobacteria	/	/	/	/	0.129	0.023
Stationary	ASV_389	Proteobacteria	Alphaproteobacteria	Rhodobacterales	Rhodobacteraceae	/	/	0.106	0.009
Stationary	OUT_423	Proteobacteria	Alphaproteobacteria	OCS116_clade	Unknown_Family	Candidatus_Phaeomarinobacter	uncultured_bacterium	0.093	0.004

Degradation	ASV_2	Proteobacteria	Gammaproteobacteria	Alteromonadales	Alteromonadaceae	/	/	0.438	0.003
Degradation	ASV_7	Firmicutes	Bacilli	Bacillales	Bacillaceae	Bacillus	/	0.207	0.001
Degradation	ASV_8	Proteobacteria	Betaproteobacteria	Burkholderiales	Burkholderiaceae	Limnobacter	uncultured_beta_proteobacterium	0.376	0.043
Degradation	ASV_10	Proteobacteria	Alphaproteobacteria	OCS116_clade	Unknown_Family	Candidatus_Phaeomarinobacter	Candidatus_Phaeomarinobacter_ectocarpi	0.173	0.003
Degradation	ASV_20	Proteobacteria	Gammaproteobacteria	Xanthomonadales	Solimonadaceae	/	/	0.919	0.002
Degradation	ASV_22	Proteobacteria	Gammaproteobacteria	Oceanospirillales	Alcanivoracaceae	Alcanivorax	uncultured_Alcanivorax_sp.	0.430	0.003
Degradation	ASV_37	Proteobacteria	Gammaproteobacteria	Thiotrichales	Piscirickettsiaceae	Methylophaga	Ambiguous_taxa	0.377	0.005
Degradation	ASV_83	Proteobacteria	Gammaproteobacteria	Oceanospirillales	Oceanospirillaceae	Pseudospirillum	Ambiguous_taxa	1.000	0.004
Degradation	ASV_331	Proteobacteria	Alphaproteobacteria	OCS116_clade	Unknown_Family	Candidatus_Phaeomarinobacter	/	0.298	0.003
Degradation	ASV_333	Bacteroidetes	Flavobacteriia	Flavobacteriales	Flavobacteriaceae	Aquibacter	/	0.229	0.033
Degradation	ASV_361	Proteobacteria	Alphaproteobacteria	OCS116_clade	Unknown_Family	Candidatus_Phaeomarinobacter	Candidatus_Phaeomarinobacter_ectocarpi	0.262	0.016
Degradation	ASV_375	Proteobacteria	Alphaproteobacteria	Rhodobacterales	Rhodobacteraceae	Marivita	/	0.391	0.039
Degradation	ASV_379	Proteobacteria	Gammaproteobacteria	Alteromonadales	Alteromonadaceae	Marinobacter	/	0.274	0.003
Degradation	ASV_407	Proteobacteria	Alphaproteobacteria	OCS116_clade	Unknown_Family	Candidatus_Phaeomarinobacter	Candidatus_Phaeomarinobacter_ectocarpi	0.440	0.015
Degradation	ASV_417	Proteobacteria	Gammaproteobacteria	Xanthomonadales	Solimonadaceae	/	/	0.857	0.002

References

1. Darzi Y, Letunic I, Bork P, Yamada T. 2018. iPath3. 0: interactive pathways explorer v3. *Nucleic Acids Res.* **46**: 510-513. https://doi.org/10.1093/nar/gky299.
2. Yao X, Zhang Y, Zhu G, Qin B, Feng L, Cai L, Gao G. 2011. Resolving the variability of CDOM fluorescence to differentiate the sources and fate of DOM in Lake Taihu and its tributaries. *Chemosphere*, **82**: 2, 145-155. https://doi.org/10.1016/j.chemosphere.2010.10.049.
3. Murphy, K.R., Stedmon, C.A., Waite, T.D., Ruiz, G.M., 2008. Distinguishing between terrestrial and autochthonous organic matter sources in marine environments using fluorescence spectroscopy. *Mar. Chem.* **108**: 40-58. https://doi.org/10.1016/j.marchem.2007.10.003.
4. Coble PG, Del Castillo, CE, Avril, B. 1998. Distribution and optical properties of CDOM in the Arabian Sea during the 1995 Southwest Monsoon. *Deep Sea Res. Part II* **45**: 2195-2223. https://doi.org/10.1016/S0967-0645(98)00068-X.
